# Telomere dysfunction promotes cholangiocyte senescence and biliary fibrosis in primary sclerosing cholangitis

**DOI:** 10.1172/jci.insight.170320

**Published:** 2023-10-23

**Authors:** Nidhi Jalan-Sakrikar, Abid Anwar, Usman Yaqoob, Can Gan, Anthony B. Lagnado, Alexander Q. Wixom, Diana Jurk, Robert C. Huebert

**Affiliations:** 1 Division of Gastroenterology and Hepatology,; 2 Gastroenterology Research Unit,; 3 Center for Cell Signaling in Gastroenterology, and; 4 Physiology and Biomedical Engineering, Mayo Clinic and Foundation, Rochester, Minnesota, USA.

**Keywords:** Gastroenterology, Hepatology, Cellular senescence, Fibrosis, Telomeres

## Abstract

Cellular senescence and biliary fibrosis are prototypical features of obliterative cholangiopathies, such as primary sclerosing cholangitis (PSC). Telomere dysfunction can lead to senescence either through telomere erosion or damaged telomeres. Our goal was to investigate a mechanistic relationship between telomere damage and biliary fibrosis in PSC. Telomere attrition was observed in the bile ducts of patients with PSC along with a reduction in telomerase reverse transcriptase (TERT) expression, compared with that in normal livers. Similarly, liver tissue from mouse models of biliary fibrosis showed telomere attrition with increased damage at telomeres measured as telomere-associated foci (TAF). Cellular models of senescence induction increased the TAF in cholangiocytes. This coincided with decreased TERT expression and increased senescence, which was rescued by modulating TERT levels. Epigenetic analysis revealed increased acquisition of repressive histone methylation at the TERT promoter, which correlated with decreased TERT transcription. Cholangiocyte-selective deletion of TERT in mice exacerbated fibrosis, whereas androgen therapy toward telomerase rescued liver fibrosis and liver function in a genetic mouse model of PSC. Our results demonstrate a mechanistic role for telomere dysfunction in cellular senescence and fibrosis that characterize PSC. This suggests that PSC may be, in part, a telomere biology disorder, and identifies TERT as a potential therapeutic target.

## Graphical abstract

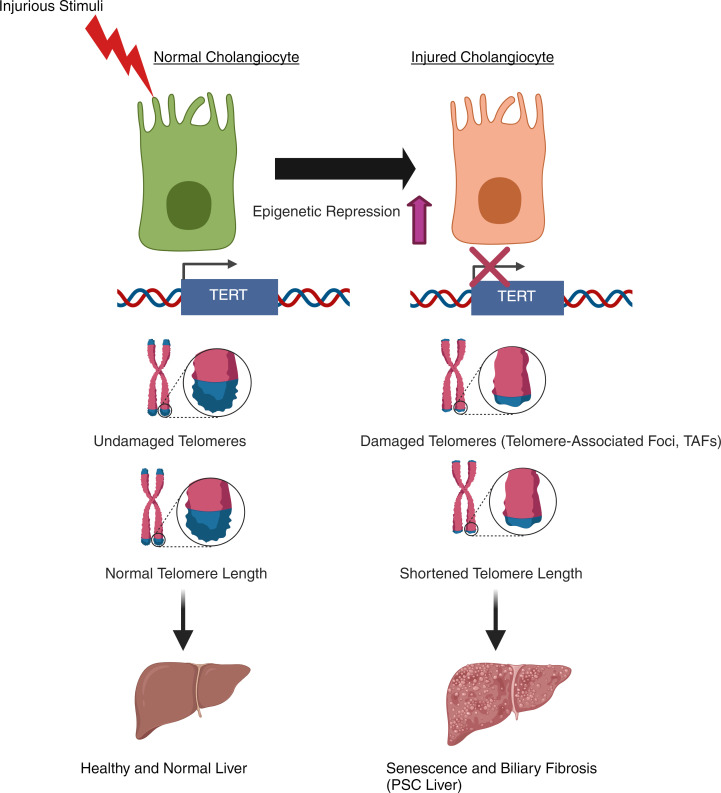

## Introduction

Primary sclerosing cholangitis (PSC) is a rare, chronic, cholestatic liver disease characterized by biliary damage that leads to strictures, fibrosis, and cirrhosis (1). Patients with PSC are also at increased risk of developing hepatobiliary malignancies, such as cholangiocarcinoma (2). No effective pharmacotherapies exist to effectively treat the disease or to delay disease progression. Liver transplantation is effective for extending the survival of patients with PSC but is not available to all patients, requires lifelong immunosuppression, and does not prevent recurrent disease after transplantation. Many studies have shown the central role of cholangiocytes (the specialized epithelial cells lining the bile ducts) in initiating and perpetuating the biliary damage in PSC (2–5). Cholangiocytes respond to the exogenous and/or endogenous insults that render them senescent and secretory such that they recruit and activate inflammatory cells and liver fibroblasts. During chronic injury, this reparative process is converted into a persistent fibroinflammatory response that perpetuates the development of biliary cirrhosis (6–8).

Cellular senescence is a state of irreversible cell cycle arrest that can be induced by a variety of stressors, including telomere dysfunction and genotoxic and oxidative stresses (9–11). Senescent cells frequently have increased secretion of a broad repertoire of proinflammatory factors, collectively known as the senescence-associated secretory phenotype (SASP), which can induce tissue dysfunction in a paracrine manner. Cellular senescence is pathogenic in several chronic conditions, including neurodegenerative disorders, obstructive pulmonary disease, and a wide range of chronic liver diseases (12). Biliary epithelial senescence is a characteristic feature of PSC that is associated with increased expression of SA-β-GAL, p16INK4A (p16), and p21WAF1/Cip1 (p21) in cholangiocytes (4, 13). Therefore, a growing body of work suggests that cellular senescence in cholangiocytes plays a key role in the pathogenesis of biliary disease (4, 5, 14).

Telomere shortening is 1 of the major inducers of cellular senescence (15–19). Telomeres are repetitive DNA sequences at the end of chromosomes. Telomere shortening occurs with each successive cell division, and if telomeres become critically short, the chromosomal ends are recognized as sites of DNA damage leading to activation of the cellular senescence program (17). Telomeric repeats are added to the chromosome via the enzyme telomerase reverse transcriptase (TERT in humans; Tert in mice). Mice lacking the Tert enzyme have telomere shortening and increased sensitivity to the liver toxin CCL4 (20). A subset of hepatocytes in mouse liver express Tert to maintain liver homeostasis, which, when genetically ablated, results in decreased liver regeneration and increased fibrosis (21). Markers of senescence and telomere-associated foci (TAF) have also been reported in liver tissues of patients with nonalcoholic fatty liver disease (NAFLD) (22). In pulmonary fibrosis, alveolar epithelial cells lacking Tert develop senescence and perpetuate the fibrotic phenomena in the lungs (23). Telomere shortening has been demonstrated in several liver diseases, including primary biliary cholangitis, NAFLD, and alcoholic liver disease (16, 18, 24–27). Although peripheral blood analysis of telomere length in patients with PSC reveals shorter telomeres (28), whether this reflects telomere dysfunction in liver tissue and in cholangiocytes has not been studied.

Here, we demonstrate progressive telomere shortening in the bile ducts of patients with PSC compared with control bile ducts. This telomere shortening is further associated with DNA damage at certain telomeres in cholangiocytes. We define a role for TERT in maintaining cholangiocyte telomere health, and we demonstrate that cholangiocyte-selective deletion of Tert in mice exacerbates diet-induced biliary fibrosis and senescence. Mechanistically, senescence induction in primary human cholangiocytes epigenetically represses TERT transcription through the enrichment of repressive trimethylation at lysine 9 of histone 3 (H3K9me3). Collectively, these studies demonstrate a role for telomere attrition in the pathogenesis of PSC and provide a rationale for telomere-directed therapeutics for treatment of PSC.

## Results

### Telomere attrition occurs in the bile ducts of PSC liver.

**Figure 1:**
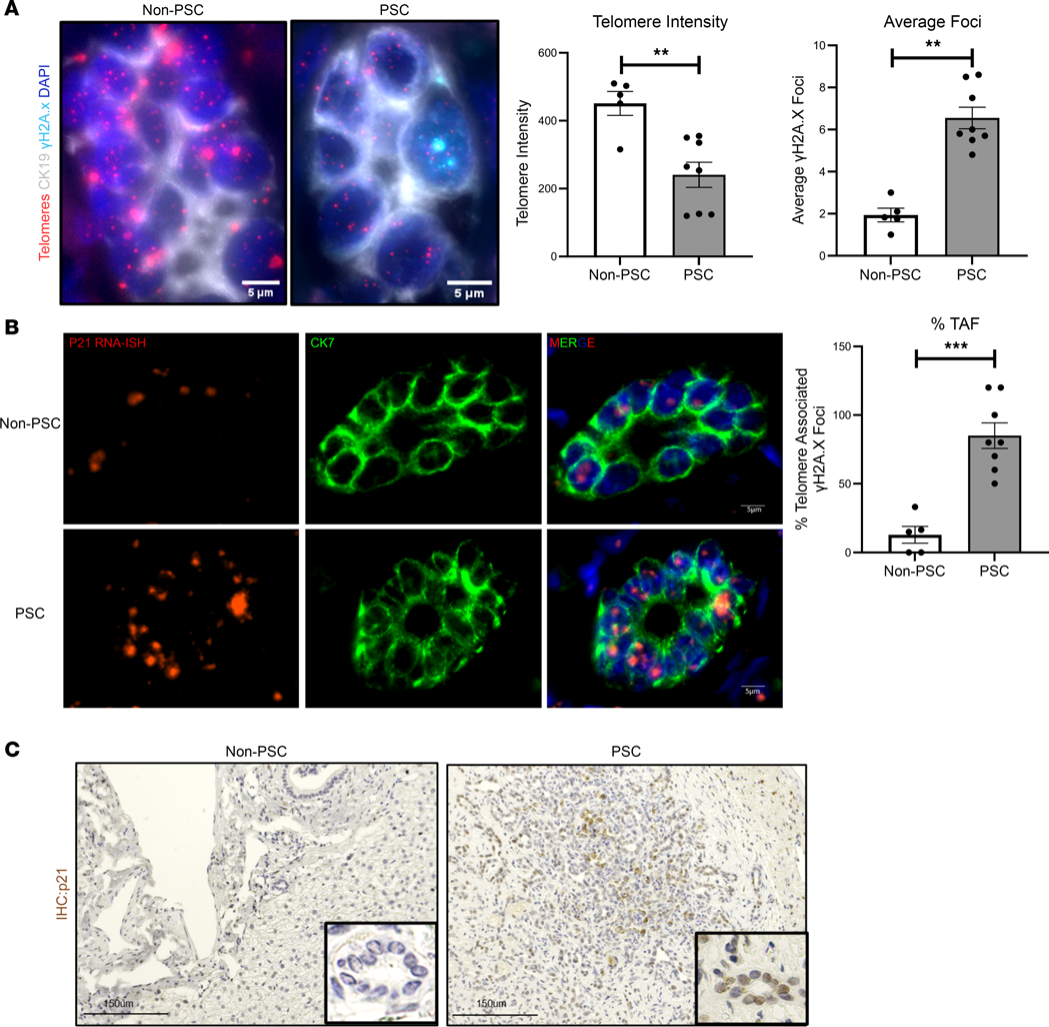
Telomere attrition occurs in the bile ducts of PSC liver. (**A**) Telomere FISH (red) with DNA damage marker γH2A.x (cyan) and cholangiocyte marker CK19 (gray) in non-PSC and PSC liver tissue. Bar graphs show quantification of telomere intensity, average γH2A.x foci, and percentage of TAF (colocalization of γH2A.x with telomeres), revealing significant decrease in telomere intensity in PSC bile ducts with increased foci and TAFs. **P < 0.001, ***P < 0.0001, unpaired, 2-tailed, *t* test. All error bars are SEM; *n* = 5–8 per group; *n* = 5–6 fields of view per patient were used for quantification. (**B**) p21 RNA ISH (red) with co-immunofluorescence for cholangiocyte marker CK7 (green) showing increased expression of p21 in PSC bile ducts; *n* = 5–8 per group. (**C**) IHC for p21 in non-PSC and PSC liver tissue, Insets show zoomed bile ducts positive for p21 (brown).

PBMC analysis demonstrated reduced telomere length in patients with PSC compared with healthy control participants (28), and senescence has been shown to be a reliable feature of the disease (13, 29). We initially aimed to investigate whether telomere attrition, in addition to senescence, can be observed in the bile ducts of patients with PSC. Telomere FISH of samples from individuals without PSC and patients with PSC revealed a significant decrease in telomere intensity (Figure 1A). In these assays, telomere intensity correlated with telomere length, and quantification demonstrated a significant decrease in telomere length (1.9-fold; *P* < 0.001 compared with normal telomere length) in cholangiocyte nuclei compared with the normal bile ducts, marked by CK19 (red spots in right-side image in Figure 1A).

Telomere dysfunction (i.e., TAF) can be associated with DNA damage at the telomeres (30). Immuno-FISH for TAF in normal and PSC liver tissue demonstrated an association of γH2A.x immunostaining with telomere FISH (Figure 1A, cyan), suggesting damage at or on the telomeres. There was a significant increase in the total number of foci and also in TAF in the bile ducts of patients with PSC (Figure 1A, right).

Telomere attrition is linked to cellular senescence in several diseases (31); therefore, we used RNA ISH for p21, a marker for cell cycle arrest, in liver tissues from normal and patients with PSC. Compared with normal liver, we noted an increase in p21 transcript in the cholangiocytes (marked by CK7) in PSC (Figure 1B). This was confirmed at the protein level by IHC (Figure 1C), suggesting cellular senescence, confirming prior studies demonstrating senescence as a feature of PSC (29). Also, p21 RNA ISH colocalized with another DNA damage marker, 53BP1, in the nuclei of PSC bile ducts (Supplemental Figure 1A; supplemental material available online with this article; https://doi.org/10.1172/jci.insight.170320DS1). Taken together, these data suggest that cellular senescence in PSC is associated with telomere attrition as well as DNA damage at the telomeres marked as TAF.

### Telomere attrition occurs in mouse models of biliary fibrosis.

**Figure 2:**
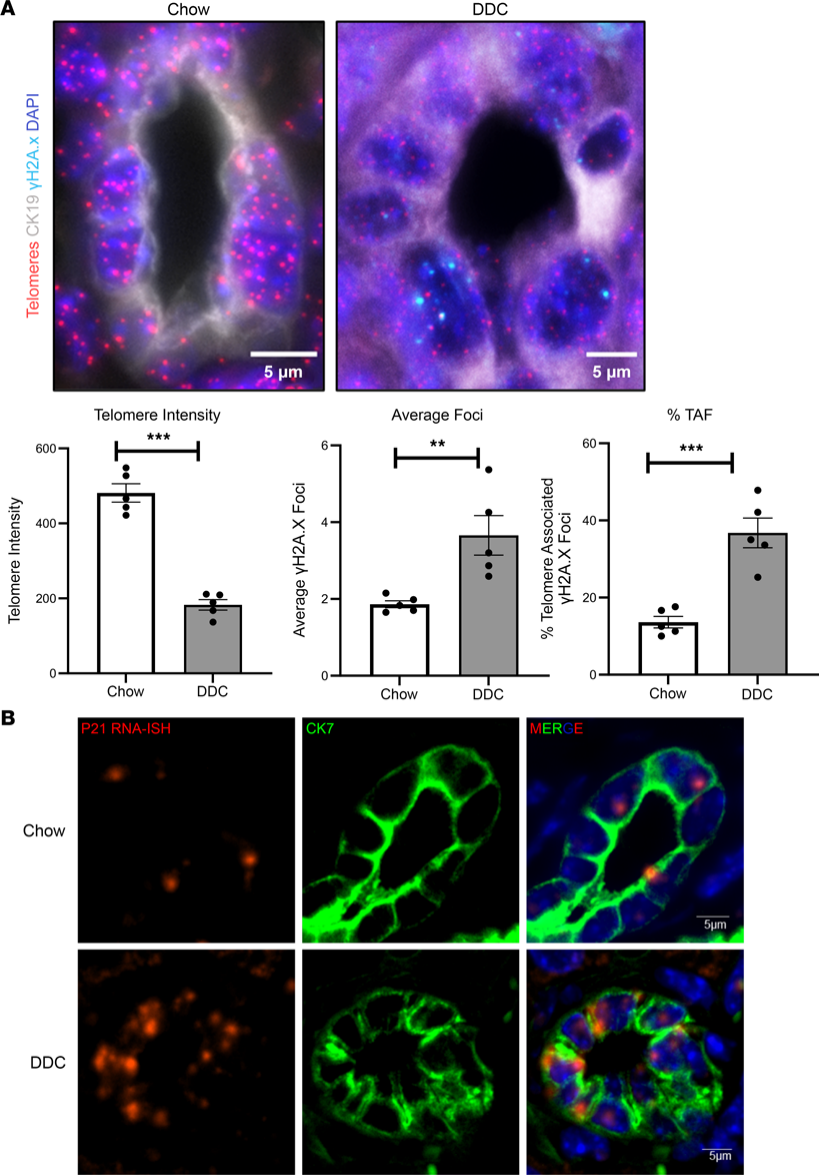
Telomere attrition occurs in mouse models of biliary fibrosis. (**A**) Telomere FISH (red) of bile ducts co-stained with CK19 (gray) and γH2A.x (cyan) from chow- and DDC-fed mice. Bar graphs show the quantification of telomere intensity, average γH2A.x foci, and %TAF demonstrating significant reduction in telomere length associated with increased DNA damage at the telomeres in DDC-injured mice. **P < 0.001, ***P < 0.0001, unpaired, 2-tailed, *t* test. All error bars are SEM; *n* = 5–6 per group; *n* = 6–7 fields of view per mouse were used for quantification. (**B**) RNA ISH for p21 transcript with immunofluorescence for cholangiocyte marker CK7 demonstrates elevated gene expression in liver tissue of DDC-fed mice compared with that of chow-fed mice.

We subsequently investigated whether mouse models of biliary fibrosis also have telomere attrition and senescence. We used liver tissues from 3,5-diethoxycarbonyl-1,4-dihydrocollidine (DDC) diet–fed mice, a model commonly used to study the pathogenesis of PSC (32). Telomere FISH revealed striking telomere attrition in bile ducts of DDC mouse livers compared with liver tissue of mice fed chow (Figure 2A). Quantification of telomere intensity decreased 3-fold in the bile ducts of the DDC-fed mouse model of biliary fibrosis (*P* < 0.0001; Figure 2A). Similar to the human tissue, costaining for γH2A.x with telomere FISH also demonstrated increased TAF in the bile ducts of DDC-fed mice (Figure 2A, bar graphs). Histograms showing distribution of the individual telomere intensities in several bile duct nuclei showed a leftward shift in the liver tissue of DDC-fed mice, suggesting an increased frequency of shorter telomeres compared with tissues of chow-fed mice (Supplemental Figure 2A).

We also performed immuno-FISH for TAF in liver tissues from bile-duct ligated mice and Mdr2*^–/–^* (Abc4*^–/–^*) mice, both of which are mouse models of biliary damage. Again, we saw telomere attrition and TAF in the cholangiocytes of these mice liver tissues (Supplemental Figure 2B). We analyzed the hepatocytes in the DDC-fed mice and observed no reduction in telomere intensity with a slight increase in DNA damage (Supplemental Figure 3A). RNA ISH for p21 transcript further showed increased expression in the bile ducts (marked by CK7) of the DDC-fed mice (Figure 2B). Altogether, these data suggest that in mouse models of chronic biliary damage, cholangiocyte senescence is associated with telomere damage and shortening.

### TERT expression is reduced in human PSC and mouse liver tissue with biliary fibrosis.

**Figure 3:**
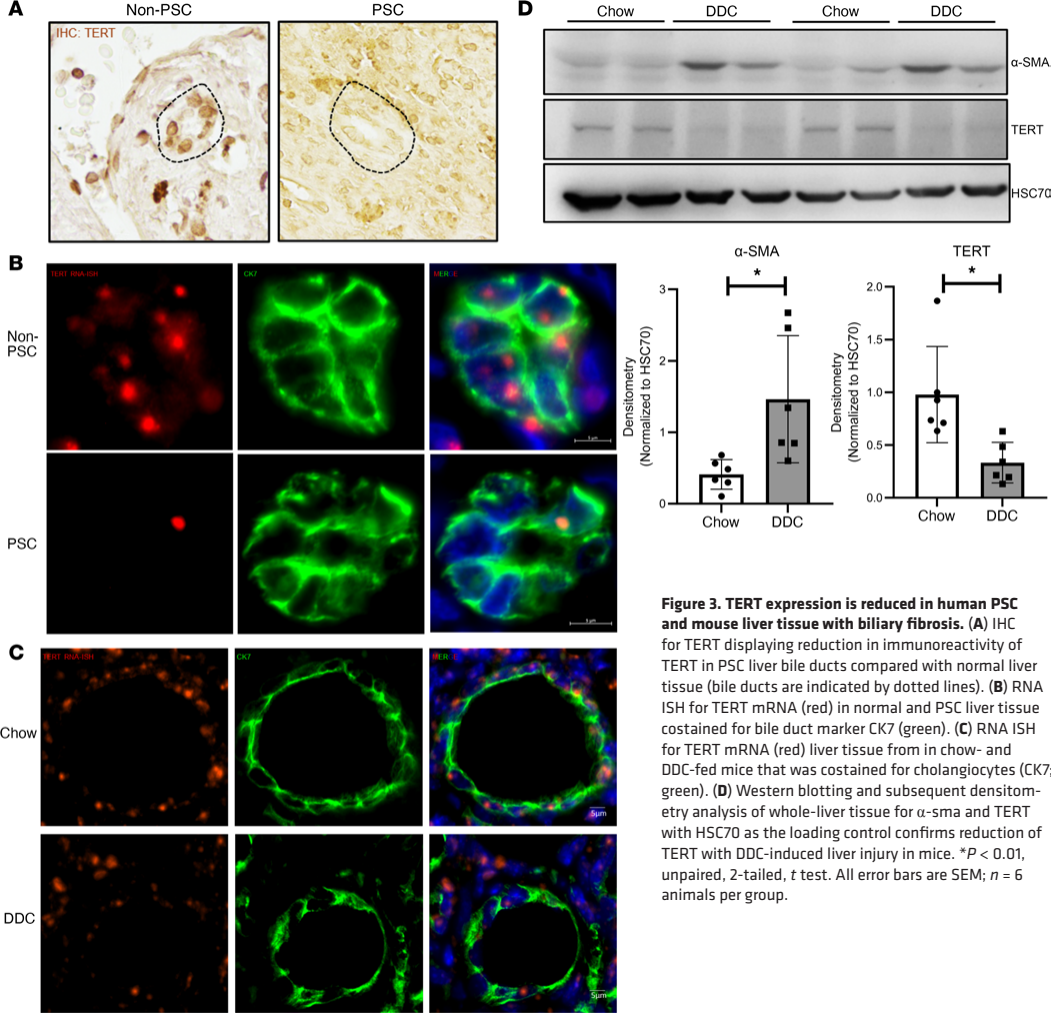
TERT expression is reduced in human PSC and mouse liver tissue with biliary fibrosis. (**A**) IHC for TERT displaying reduction in immunoreactivity of TERT in PSC liver bile ducts compared with normal liver tissue (bile ducts are indicated by dotted lines). (**B**) RNA ISH for TERT mRNA (red) in normal and PSC liver tissue costained for bile duct marker CK7 (green). (**C**) RNA ISH for TERT mRNA (red) liver tissue from in chow- and DDC-fed mice that was costained for cholangiocytes (CK7; green). (**D**) Western blotting and subsequent densitometry analysis of whole-liver tissue for α-sma and TERT with HSC70 as the loading control confirms reduction of TERT with DDC-induced liver injury in mice. *P < 0.01, unpaired, 2-tailed, *t* test. All error bars are SEM; *n* = 6 animals per group.

TERT is the catalytic enzyme that is indispensable for the maintenance of telomere length and integrity. We investigated whether TERT is expressed in human liver tissues and if the levels are altered in PSC. IHC for TERT in normal human liver, indeed, showed nuclear staining, with immunoreactivity in the bile ducts. However, in PSC liver tissues, we observed a decrease in the immunoreactivity for TERT (Figure 3A, bile ducts marked by dotted lines). This phenomenon was also confirmed by RNA FISH for TERT transcript in the bile ducts (marked by CK7), where we found decreased TERT transcript levels (Figure 3B, red).

We then measured the mRNA and protein expression of TERT in the liver of control and fibrotic mice. RT-PCR analysis demonstrated significant reduction in TERT mRNA in whole livers from DDC-fed mice (Supplemental Figure 3B). Mdr2*^–/–^* mice also display features of PSC (senescence and fibrosis) as they age. Accordingly, we saw a similar decrease in TERT expression in livers of Mdr2*^–/–^* mice (Supplemental Figure 3B). RNA ISH for TERT transcript in mouse liver bile ducts (marked by CK7) were positive for TERT, which was decreased with DDC diet, corroborating the mRNA findings (Figure 3C). Western blotting for TERT protein also validated the mRNA observations (Figure 3D) by confirming that the DDC-fed mice had reduced TERT protein levels in liver tissue, with an increase in fibrosis marker α-smooth muscle actin (α-sma). Collectively, these data suggest that the cholangiocytes of patients with PSC undergo a cascade of pathological events, including reduced TERT levels, DNA damage, telomere attrition, and, ultimately, cellular senescence leading to fibrosis.

### Cellular models of senescence reduce TERT in cholangiocytes.

**Figure 4:**
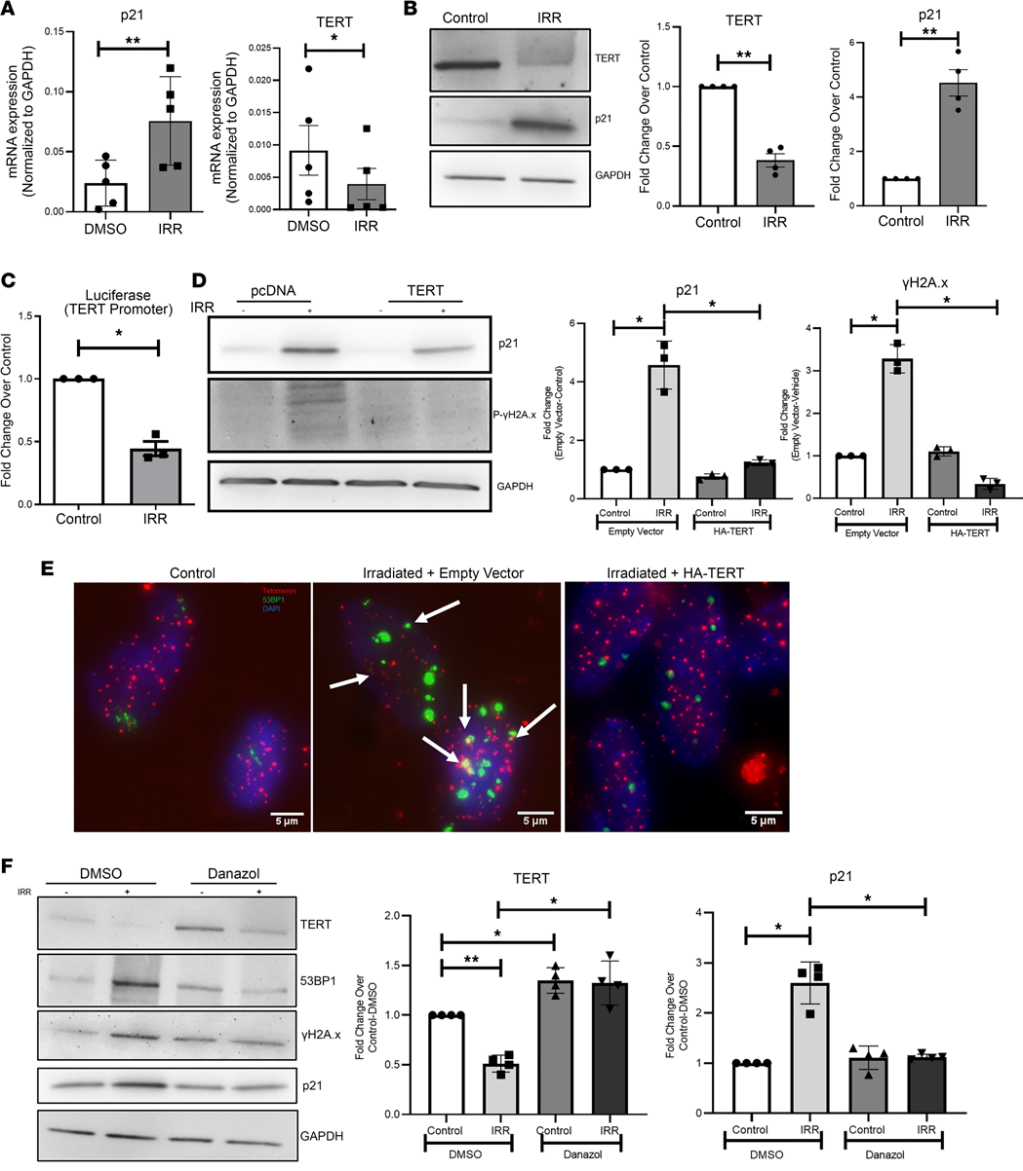
Cellular models of senescence reduce TERT in cholangiocytes. (**A**) RT-PCR analysis of HiBECs shows an approximately 3-fold increase in p21 gene transcription and a 0.5-fold decrease in the TERT transcript in irradiated (IRR) cells compared with control cells. *P < 0.01, **P < 0.001, paired, 2-tailed *t* test. All error bars are SEM; *n* = 5. (**B**) IB of p21 and TERT with GAPDH as the loading control in HiBECs 6 days after irradiation. Densitometry data reported in the bar graphs show increased p21 with a decrease in TERT upon irradiation. **P < 0.001, paired, 2-tailed *t* test. All error bars are SEM; *n* = 4. (**C**) Luciferase assay revealing decrease in TERT transcription in irradiated cells compared with controls cells transfected with luciferase-fused TERT promoter. *P < 0.01. All error bars are SEM; *n* = 3. (**D**) IB of HiBECs overexpressing TERT reveals a decrease in irradiation-induced p21 and γH2A.x. Densitometry demonstrates TERT overexpression preventing irradiation-induced DNA damage and increase in p21 expression. *P < 0.01. All error bars are SEM; *n* = 3. (**E**) Immuno-FISH for telomeres (red) and the DNA damage marker 53BP1 (green) in primary cholangiocytes reveals reduced DNA damage in cells transfected with TERT upon irradiation. (**F**) Western blotting of HiBECs exposed to irradiation showing rescue of TERT levels by danazol and reduced levels of DNA damage markers 53BP1 and γH2A.x, as well as of p21 compared with DMSO-treated cells with GAPDH as a loading control. Densitometry graphs confirm the increase in TERT levels with a concomitant decrease in the senescence marker p21. *P < 0.01, **P < 0.001, 1-way ANOVA followed by Tukey’s post-test. All error bars are SEM; *n* = 4.

We used human intrahepatic biliary epithelial cells (HiBECs) in vitro to further investigate the role of TERT in cholangiocytes. HiBECs were irradiated to induce senescence. Six days after irradiation, we used a senescence microarray to understand gene expression changes. We observed an increase in levels of the senescence marker p21 (CDKN1A gene) as well as CDKN2B, with a concomitant decrease in TERT expression in these cells (Supplemental Figure 4, A and C). RT-PCR analysis further confirmed that TERT levels were reduced both at the mRNA (Figure 4A) and protein (Figure 4B) levels, with a corresponding increase in p21 levels with irradiation in cholangiocytes. We also corroborated these findings in other in vitro models of senescence, such as LPS and TGF-β treatment (Supplemental Figure 4, B, D, and E).

To determine whether TERT repression is occurring at the transcriptional level, we used luciferase assays. HiBECs transfected with luciferase-fused TERT promoter were exposed to irradiation. Compared with control cells transfected with TERT promoter, TERT transcription was significantly decreased in irradiated cells (Figure 4C).

To understand whether restoration of TERT levels could rescue the senescence phenotype, we overexpressed TERT in HiBECs and then exposed them to irradiation. RT-PCR confirmed a 60-fold overexpression of TERT compared with an empty vector control (Supplemental Figure 4F). This was sufficient to block the irradiation-induced increase in p21 in HiBECs (Supplemental Figure 4F and Figure 4D). IB for γH2A.x also revealed a lack of DNA damage induction upon TERT overexpression (Figure 4D).

Next, we measured TAF in our primary cholangiocytes exposed to irradiation. We observed little to no staining for the DNA damage marker 53BP1 in control cells. In contrast, cells exposed to irradiation had increased foci for DNA damage 48 hours later (Figure 4E).

We next asked whether the presence of TERT is sufficient to prevent the acquisition of DNA damage at the telomeres. We transfected cholangiocytes with TERT and then exposed the cells to irradiation. Cells were fixed and stained for 53BP1 and telomeres 48 hours later. With TERT overexpression, 53BP1 staining at the telomeres was abrogated (Figure 4E, right-most image).

Danazol, a sex hormone, has been shown in clinical trials to increase telomere length through increasing TERT expression and to improve disease phenotype in patients with telomere biology disorders (33). To test the applicability of danazol in biliary senescence, we treated primary cholangiocytes with danazol after exposure to irradiation. Under control (DMSO) conditions, there was a decrease in TERT levels, which was rescued by danazol (Figure 4F). Along with a rescue of TERT levels, we also observed a decrease in the DNA damage markers, γH2A.x and 53BP1. These biochemical studies in HiBECs indicate that restoration of TERT levels can maintain the physiological health of telomeres in cholangiocytes.

### TERT modulation regulates TGF-β–mediated effects on cholangiocytes.

Because TGF-β is significantly increased in PSC and is profibrotic in the disease (8), we investigated the mechanistic role of TERT in mediating the downstream effects of TGF-β. Our previous studies have shown that TGF-β treatment in H69 cells increases the release of fibrogenic molecules such as fibronectin (FN1) and plasminogen activator inhibitor-1 (PAI-1) (7).

**Figure 5:**
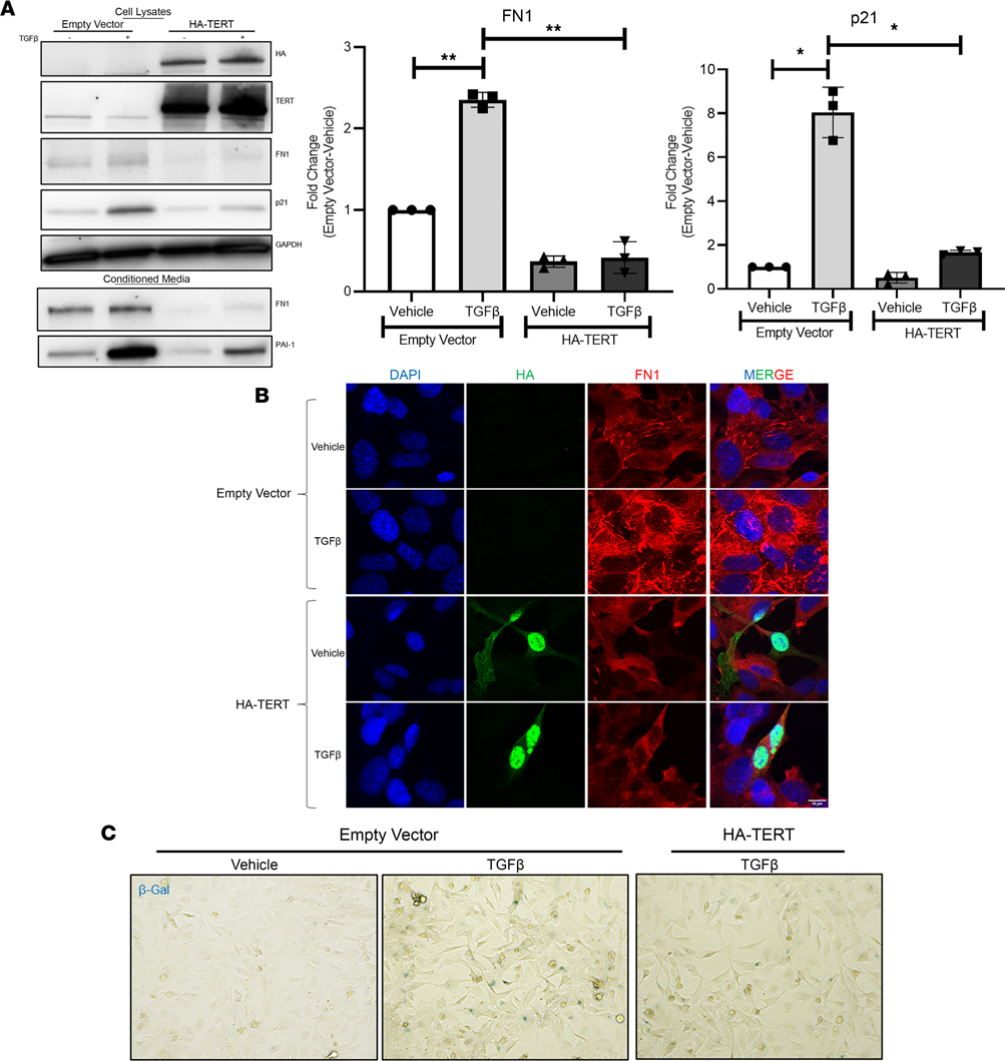
TERT attenuates the TGF-β*–*mediated release of fibrogenic molecules. (**A**) Protein lysates from H69 cells treated with vehicle or 10 ng/mL TGF-β in the presence of HA-TERT plasmid or an empty vector were immunoblotted for HA, TERT, FN1, p21, and GAPDH (loading control). Conditioned medium was blotted for released FN1 and PAI-1 (lower gel image). Densitometric quantification reveals protein levels of FN1 and p21 were reduced upon HA-TERT overexpression compared with empty vector control cells (bar graphs). *P < 0.01, **P < 0.001, 1-way ANOVA followed by Tukey’s post-test. All error bars are SEM; *n* = 3. (**B**) Immunofluorescence for HA and FN1 in H69 cholangiocytes displays significantly reduced levels of TGF-β*–*induced FN1 in HA-TERT transfected cells. (**C**) β-Gal staining of H69 cells transfected with HA-TERT or empty vector and treated with TGF-β demonstrated reduced staining in the presence of TERT.

We transfected H69 cells with control plasmid or plasmid encoding HA-tagged TERT (HA-TERT) and then treated them with either vehicle or TGF-β. mRNA analysis showed a 4-fold increase in FN1, a known paracrine activator of hepatic stellate cells. This occurred in conjunction with a 3-fold increase in p21 after TGF-β treatment. These effects were significantly blunted in the presence of TERT overexpression as assessed by RT-PCR (Supplemental Figure 5A). Western blotting further confirmed the overexpression of TERT and decrease in TGF-β–induced protein expression of FN1 and p21 (Figure 5A, densitometry results in bar graphs). Additionally, conditioned medium from the cholangiocytes also had reduced secretion of FN1 and PAI-1 from cells overexpressing TERT (Figure 5A, lower gel image). We also tested for a proinflammatory cytokine, CCL2, by RT-PCR and ELISA, which demonstrated decreased expression and release in presence of HA-TERT (Supplemental Figure 5, A and B). Cytokine array analysis on the medium further demonstrated a decrease in levels of several other cytokines, including MIC-1, IL-8, and VEGF (Supplemental Figure 5C). Immunofluorescence for HA in the transfected cells showed nuclear localization of the transfected HA-TERT.

FN1 staining demonstrated an increase with TGF-β, which was attenuated in the presence of HA-TERT (Figure 5B). β-Galactosidase (β-gal) staining assay further confirmed the presence of senescent cells (blue in Figure 5C) with TGF-β treatment, which was decreased upon TERT overexpression. Next, we isolated primary cholangiocytes from mice and treated them with TGF-β for 24 hours. mRNA and protein analysis of the cells confirmed a decrease in Tert levels with increased expression of fn1 and pai-1 (Supplemental Figure 5, D and E). We also observed a decrease in the proliferation marker ki-67 upon TGF-β treatment (Supplemental Figure 5E).

To test the specificity of the TERT effect in cholangiocytes, we isolated mouse primary hepatocytes and treated them with TGF-β. Even though we observed an increase in fn1 protein expression with TGF-β in hepatocytes, we did not observe a decrease in Tert levels nor an increase in p21 (Supplemental Figure 5F). This was consistent at the gene expression level as measured by RT-PCR analysis (Supplemental Figure 5G). Interestingly, when we isolated intrahepatic leukocytes from mice fed chow or DDC, RT-PCR analysis revealed a decrease in Tert expression upon injury with no increase in p21 expression, suggesting a different role for tert in these cells (Supplemental Figure 5H). Overall, these data demonstrate that TERT can suppress senescence and the associated fibrogenic phenotype in cholangiocytes.

Because TERT overexpression prevented cholangiocytes from secreting fibrogenic molecules in response to TGF-β, we next sought to assess whether loss of TERT would exacerbate TGF-β*–*mediated effects in cholangiocytes. siRNA-mediated TERT knockdown was confirmed by RT-PCR and Western blotting (Supplemental Figure 6, A and B). Silencing of TERT significantly increased the TGF-β*–*induced FN1 and p21 levels compared with control cells (Supplemental Figure 6, A and B). Furthermore, in contrast to our overexpression model, protein levels of FN1 and PAI-1 were increased in the bioactive secretome of TERT-siRNA–treated cells compared with control cells.

To demonstrate the effect of pharmacological inhibition in human cholangiocytes, we used the small-molecule telomerase inhibitor BIBR 1532, which reduces hTERT expression and inhibits the activity of telomerase by targeting core components of telomerase in human cells (34). Cholangiocyte cells were treated with BIBR 1532 or DMSO (control) before treatment with TGF-β. Consistent with our genetic knockdown findings, pharmacological inhibition of TERT with BIBR 1532 and TGF-β led to significant exacerbation of FN1 and p21 protein levels (Supplemental Figure 6C). Together, these results suggest that TERT inhibition intensifies the TGF-β*–*dependent induction of fibrogenic and senescent phenotypes in human cholangiocytes.

### TERT is epigenetically repressed in senescent cholangiocytes.

**Figure 6:**
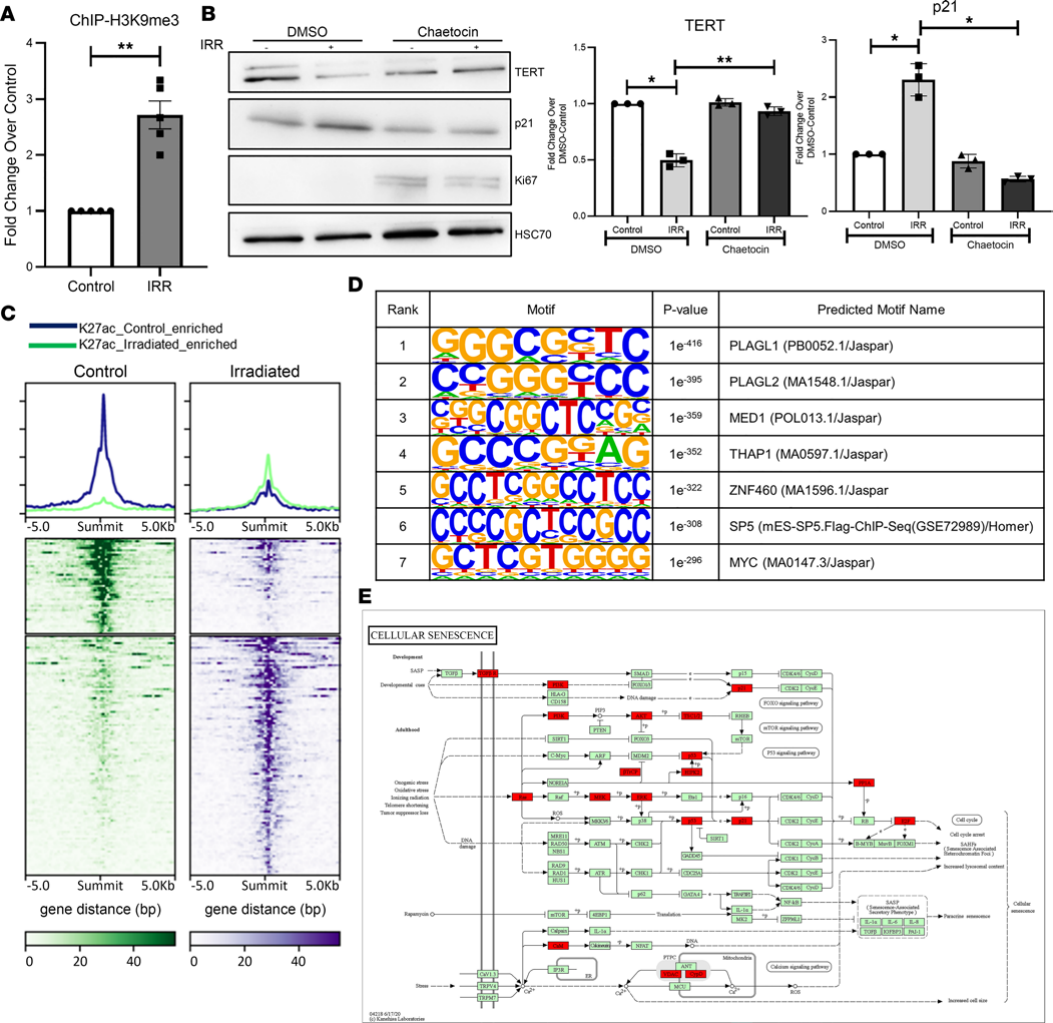
Epigenetic regulation of TERT modulates cholangiocyte senescence. (**A**) ChIP analysis for H3K9me3 at the TERT promoter reveals increased occupancy in cholangiocytes exposed to irradiation (IRR). **P < 0.01, paired, 2-tailed *t* test. All error bars are SEM; *n* = 3. (**B**) IB in cholangiocytes for TERT, p21, Ki-67, and HSC70 (loading control) treated with chaetocin or DMSO. Densitometry data (bar graphs) reveal reduced p21 protein levels with irradiation upon treatment with chaetocin, which increases TERT expression. *P < 0.01, **P < 0.001, 1 way ANOVA followed by Tukey’s post-test. All error bars are SEM; *n* = 3. (**C**) ChIP-Seq for H3K27ac in control and irradiated cholangiocytes showing gain in new sites upon irradiation (green). (**D**) Motif enrichment analysis of regions gaining occupancy of H3K27ac with irradiation reveals senescence-associated transcription factors. (**E**) Kyoto Encyclopedia of Genes and Genomes (KEGG) pathway analysis of genes enriched with H3K27ac shows senescence-related pathways.

Because we observed a reduction in TERT protein and gene expression levels in senescent cholangiocytes accompanied by reduced transcription, we asked whether this could be due to epigenetic repression. In neurons, H3K9me3 results in TERT repression (35). In cholangiocytes, ChIP analysis, indeed, showed an approximately 3-fold increase in H3K9me3 at the TERT promoter upon irradiation (Figure 6A). Then, we used an H3K9me3 inhibitor, chaetocin, in combination with irradiation in cholangiocytes. Compared with DMSO-treated cells, chaetocin treatment rescued the irradiation-induced reduction in TERT levels. This rescue in TERT was sufficient to reduce senescence and increase proliferation in the cholangiocytes (Figure 6B).

To gain more insight into the epigenetic regulation of senescence in cholangiocytes, we performed ChIP-Seq for H3K27ac on nonirradiated (control) and irradiated cholangiocytes. There was an increase of H3K27ac acquisition in a distinct set of gene promoters (Figure 6C, green line). Conversely, a smaller proportion of genes enriched in H3K27ac in control cells had a reduction of the mark in the irradiated condition (Figure 6C, blue line). Motif analysis on the sites gaining H3K27ac during irradiation revealed transcription factor binding sites such as PLAGL1, THAP1, SP5, and MYC (Figure 6D), all of which play an important role in mediating senescence (36–38). These data suggest a gain in the senescence-associated transcriptional network during irradiation of cholangiocytes, further confirmed by Kyoto Encyclopedia of Genes and Genomes (KEGG) pathway analysis finding a significant portion of the cellular senescence pathway accumulating the activating acetylation mark (Figure 6E, genes are colored red).

### TERT deletion in bile ducts exacerbates fibrosis in mice.

To determine whether the absence of TERT in cholangiocytes is pathological in vivo, we created 2 transgenic mouse lines harboring Cre-inducible cholangiocyte-selective deletion of Tert. To do so, we crossed Tert*^fl/fl^* mice with mice harboring 2 different Cre-drivers for cholangiocytes; Krt19Cre^ERT2^-RFP and OpnCre^ERT2^. IHC for opn protein in liver tissue from the Cre^–^ and Cre^+^ mice after tamoxifen injections demonstrated the specificity for bile ducts (Supplemental Figure 7A). Mice aged 5–6 weeks were injected with tamoxifen to induce Cre expression and delete Tert in cholangiocytes. After a 1-week tamoxifen washout period, mice were used either for primary cholangiocyte isolation or placed on chow diet (control) or the DDC diet for 3 weeks. Primary cholangiocytes isolated from the Tert*^fl/fl^*/opnCre^ERT2^ mice tissue had reduced Tert expression, with a basal increase in p21 and fn1 protein expression (Supplemental Figure 7B). Analysis of the conditioned medium of the primary cholangiocytes from these mice further showed an increase in release of fn1 and pai-1 (Supplemental Figure 7B).

**Figure 7:**
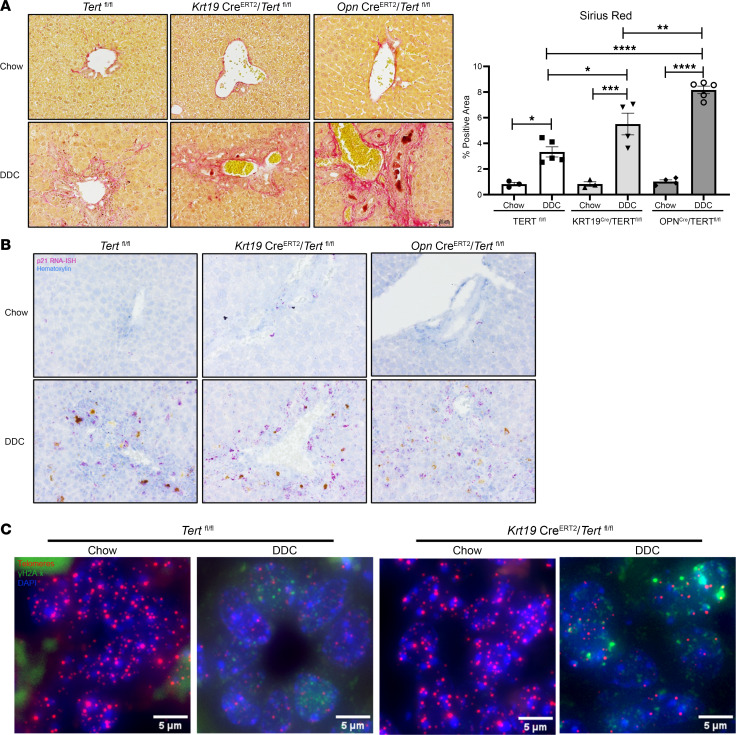
TERT deletion in bile ducts exacerbates fibrosis in mice. (**A**) Picrosirius red staining images of tissue from chow- and DDC-fed mice revealing exacerbation of fibrosis in mice with TERT deletion. Bar graph displays the quantification for the percent positive area for Picrosirius red. *P < 0.01, **P < 0.001, ***P < 0.0001, ****P < 0.00001, 1 way ANOVA followed by Tukey’s post-test. All error bars are SEM; *n* = 4–5 animals/group. (**B**) RNA ISH for p21 in chow- and DDC-fed mice show amplified levels of p21 transcript with DDC diet in mice lacking TERT. (**C**) Immuno-FISH for telomeres and γH2A.x in mice fed chow or DDC diet shows increased DNA damage in mice lacking tert compared with tert^+^ mice.

Mice fed chow or the DDC diet were weighed once a week for the duration of the experiment. As expected, control mice lost weight with the DDC diet over 3 weeks compared with those fed the chow diet. This weight-loss was further exacerbated upon Tert deletion (Supplemental Figure 7C). Picrosirius red (Figure 7A) and trichrome staining (Supplemental Figure 8A) assessment also demonstrated worsened fibrosis in the DDC-fed mice with Tert deletion compared with the control mice fed the DDC diet. mRNA analysis showed increase in gene expression for markers of liver fibrosis (col1A1 and α-sma) (Supplemental Figure 8B). RNA ISH analysis for the senescence marker p21 also showed an increase in p21^+^ cells with the DDC diet, which was further exacerbated in the Tert-deleted mice (Figure 7B). Immuno-FISH for TAF demonstrated increased DNA damage at the telomeres in the DDC-fed mice with tert deletion compared with that in tert^+^ mice (Figure 7C). These data suggest a critical role for telomere maintenance via TERT in cholangiocytes.

### Pharmacological therapy ameliorates liver injury in Mdr2^–/–^ mice.

**Figure 8:**
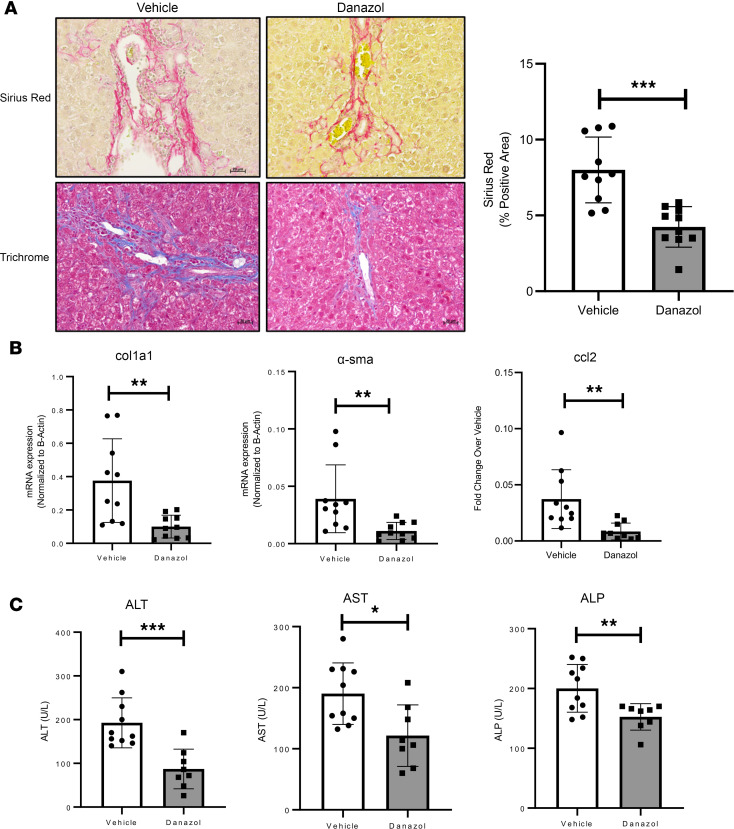
Danazol treatment improves fibrosis in Mdr2*^–/–^* mice. (**A**) Picrosirius red and trichrome staining of liver tissues from vehicle- and danazol-treated MDR2*^–/–^* mice. Bar graph displays the quantification for the percent positive area for Picrosirius red. ***P < 0.0001, unpaired, 2-tailed, *t* test. All error bars are SEM; *n* = 9–10 animals/group; *n* = 4–5 fields of view per animal were used for quantification. (**B**) RT-PCR of whole-liver tissue indicates decreased gene expression of col1a1, α-sma, and ccl2 in danazol-injected Mdr2*^–/–^* mice. *P < 0.01, **P < 0.001, unpaired, 2-tailed, *t* test. All error bars are SEM; *n* = 8–10 animals/group. (**C**) Serum analysis for liver biochemistry reveals reduced levels of alanine transaminase (ALT), aspartate aminotransferase (AST), and alkaline phosphatase (ALP) in MDR2*^–/–^* mice administered danazol compared with vehicle-treated mice. *P < 0.01, *P < 0.001. ***P < 0.0001, unpaired, 2-tailed, *t* test. All error bars are SEM; *n* = 8–10 animals/group.

Because in vitro treatment of cholangiocytes with danazol attenuated irradiation-induced damage (Figure 4F), we also investigated whether danazol treatment in vivo can attenuate the biliary fibrosis seen in Mdr2^–/–^ mice. Six-week-old Mdr2^–/–^ mice were administered daily i.p. injections for 5 weeks with either vehicle or danazol. Sirius red and trichrome staining demonstrated reduced collagen deposition and attenuated fibrosis in the danazol-treated mice (Figure 8A). RT-PCR analysis of liver tissue also showed reduced hepatic expression of fibrosis markers col1A1 and α-sma (Figure 8B). We also found reduced expression of proinflammatory cytokine CCl2 with danazol (Figure 8B). Additionally, liver biochemistries from mice serum demonstrated improvement in alanine transaminase, aspartate aminotransferase, and alkaline phosphatase levels (Figure 8C). Collectively, these findings indicate telomerase-directed pharmacological treatment can play a pivotal role in attenuating the fibroinflammatory phenotype.

## Discussion

It is increasingly recognized that cellular senescence is a characteristic feature of diseased cholangiocytes (3, 14, 29). Furthermore, it is becoming clear that the secretory phenotype that accompanies senescence contributes to biliary fibrosis through epithelial and mesenchymal crosstalk and paracrine activation of hepatic stellate cells and portal fibroblasts (8). However, important gaps still exist in our understanding of the underlying pathophysiologic mechanisms driving senescence and fibrosis in biliary diseases, such as PSC. In this study, we aimed to elucidate a novel, mechanistic relationship among cholangiocyte telomere dysfunction, DNA damage, cellular senescence, and biliary fibrosis. In this context, we report several novel, to our knowledge, advances: (a) significant telomere attrition is evident in the cholangiocytes of human PSC liver and in mouse models of biliary fibrosis; (b) expression of TERT, the enzyme responsible for telomere maintenance, is attenuated in human PSC liver, in mouse models of biliary fibrosis, and in cellular models of senescence; (c) TERT overexpression attenuates the TGF-β*–*mediated release of fibrogenic molecules, whereas TERT silencing exacerbates this process; (d) TERT is epigenetically repressed in senescent cholangiocytes through deposition of the H3K9me3 silencing mark in its promoter; (e) selective genetic deletion of Tert from mouse cholangiocytes exacerbates biliary fibrosis; and (f) androgen therapy in a mouse model of PSC improves fibrosis and liver function.

Our study findings corroborate the link between cellular senescence and a profibrogenic secretome. The study also elucidates a role for epigenetic TERT repression and telomere attrition as a driver of DNA damage, cellular senescence, and fibrosis in cholestatic liver diseases, such as PSC. Collectively, the results suggest that PSC may be, in part, a telomere biology disorder, and that epigenetic interventions aimed at preventing TERT repression and telomere loss may be rational therapeutic strategies.

Conservation of functional telomeres is vital for genomic stability and cell survival. Telomeres are composed of the tandemly repeated hexanucleotide TTAGGG in the leading 5′-strand of the chromosome. However, as humans age, somatic cells fail to express sufficient levels of TERT, which thus can result in telomere erosion and, ultimately, replicative senescence and apoptosis (15). Inactivating germline mutations in telomerase genes are associated with liver cirrhosis in humans and mice (20, 39, 40). It has also been noted that a subpopulation of hepatocytes with high telomerase expression in the liver lobule maintains liver homeostasis and protects from injury-induced fibrosis (21). Our study’s observations in cholangiocytes align well with the hepatocyte model and other reported roles on TERT-based therapy in inflammatory, fibrotic, and degenerative disorders (20, 23, 35, 41, 42).

In previous studies, the PBMCs of patients with PSC had decreased telomere length and more rapid telomere attrition, indicative of overall telomere dysfunction (28). Additionally, PSC cholangiocytes exhibit SASP markers as well as a profibrotic phenotype (29). Primary biliary cirrhosis (PBC) is an autoimmune liver disorder similar to PSC, with cholestasis and fibroinflammatory damage of the small bile ducts (18). In PBC, cellular senescence of bile ductules is also associated with telomere shortening and DNA damage (18). Our study demonstrates a mechanistic relationship between trimethylation of H3K9 within the TERT promoter to the eventual fibrosis and senescence that is characteristic of PSC. Telomere dysfunction and the resulting senescence are prominent characteristics of aging in mammals (43). Methods to attenuate senescence by restoration of telomerase activity lead to reversal of tissue degeneration in aged mice (41). Additionally, use of adeno-associated virus serotype 9 (AAV9) vectors to ectopically express TERT in adult mice had beneficial effects such as decreased glucose intolerance, reduced incidence of age-related osteoporosis, improved neuromuscular function, and longevity (44). However, a potential drawback of the ectopic expression of TERT is the development of immortal proliferative properties (i.e., cancer), because lengthening telomeres allows cells to bypass senescence (45). Despite this, AAV9–mouse TERT–treated mice did not exhibit increased tumorigenesis compared with control mice treated with AAV9-eGFP (44). TERT has also been demonstrated to have functions beyond telomere lengthening. For example, TERT can form complexes with transcription factors and chromatin remodelers to modulate cell signaling pathways (46, 47). Interestingly, TERT can also modulate mitochondrial DNA and protect against oxidative stress–induced damage (48). The telomere dysfunction in cholangiocytes, identified here, also raises the intriguing question of whether PSC represents features of premature aging of the biliary tree and whether emerging therapies in longevity science may be applicable.

Androgen therapy in patients with telomere biology disorders leads to telomere elongation in leukocytes and improved hematologic outcomes (33). Whether a similar therapeutic approach could be used for the treatment of liver diseases remains unknown, but low levels of androgen have been identified in liver diseases such as NAFLD (49). Indeed, there appears to be telomere dysfunction during NAFLD in both mice and humans (26, 50). Our mice studies demonstrate the potential of androgen therapy in improving liver function in the context of biliary fibrosis. Telomere shortening is also a general marker of, and contributor to, the pathogenesis of human liver cirrhosis (20, 39). Adenoviral reactivation of the telomerase gene in telomerase-deficient mice alleviates liver fibrosis and improves liver function (20). Congruently, our in vivo work here shows that cholangiocyte-selective KO of TERT exacerbates biliary fibrosis.

In conclusion, we have demonstrated that the cellular senescence that occurs in biliary epithelial cells during PSC is mechanistically driven by epigenetic repression of TERT, telomere attrition, and accumulation of DNA damage. These events are key drivers of the fibrosis that perpetuates the disease and leads to end-stage organ dysfunction. Correspondingly, our hope is that epigenetic pharmacology or RNA therapeutics can now be used to target telomere dysfunction selectively in cholangiocytes to slow, prevent, or even reverse biliary fibrosis.

## Methods

### Cell culture and treatments.

The H69 cholangiocyte cell line was provided by N. LaRusso (Mayo Clinic, Rochester, Minnesota, USA). HiBECs were purchased from ScienCell (catalog 5100). Normal human cholangiocytes (NHCs) were provided by Jesus Banales (Biodonostia Institute, San Sebastian, Spain). Cells were grown in DMEM/F12 supplemented with 10% FBS, 1% penicillin/streptomycin, adenine, insulin, epinephrine, T3-T, hydrocortisone, and EGF. Cells were serum starved in basal DMEM containing 1% penicillin/streptomycin for 4 hours or overnight before treatment with 10 ng/mL recombinant TGF-β (R&D Systems, catalog 240-B). Senescence was induced either by exposing the cells to X-ray irradiation (20 Gy) and maintaining in culture for 5 days, with medium change at 48 hours, or by treating cells with 10 ng/mL TGF-β for 24 hours after 4 hours of serum starvation in basal DMEM containing 1% penicillin/streptomycin. LPS-induced senescence was obtained by exposing the cholangiocytes to 200 ng/mL LPS for 10 days (changing the medium with fresh LPS every alternate day), as previously described (3). Danazol, BIBR1532, and chaetocin were purchased from Selleckchem (catalog S9506, S1186, and S8068, respectively) and dissolved per the manufacturer’s suggestion. All drug treatments were done before the senescence induction. HA-TERT and TERT promoter fused to luciferase were purchased from Addgene (catalog 51637 and 84924, respectively). TERT siRNA smartpool was from Horizon Discovery (catalog L-003547-00-0005). For overexpression, Lipofectamine 3000 (Thermo Fisher Scientific) was used, and for siRNA, DharmaFECT (catalog T-2001-01, Horizon Discovery) was used.

### Animal models.

Krt19-Cre^ERT^-LSLTdTomato (Krt19-Cre) mice were obtained from Stuart Forbes (University of Edinburgh, Edinburgh, United Kingdom). Osteopontin-Cre^ERT2^ (Opn-Cre) mice were obtained from Paul Monga (University of Pittsburgh, Pittsburgh, Pennsylvania, USA). Tert*^fl/fl^* mice were obtained from Sem Phan (University of Michigan School of Medicine, Ann Arbor, Michigan, USA). These mice were crossed to obtain Krt19Cre^ERT^/Tert*^fl/fl^* or OpnCre^ERT2^/Tert*^fl/fl^* offspring. At 5–6 weeks of age, Tert*^fl/fl^* (control), Krt19Cre^ERT^/Tert*^fl/fl^*, or Opn Cre^ERT2^/Tert*^fl/fl^* (experimental) mice (males and females) were injected with tamoxifen (20 mg/kg in corn oil) for 5 days. After a washout period of 1 week, the mice were placed on a 0.1% DDC diet (Dyets Inc.) for 3 weeks. The mice were weighed once every week for the duration of the experiment. Three weeks later, animals were sacrificed. Liver tissue was harvested for mRNA and protein and then was paraffin embedded for histology. Fibrosis was analyzed by Sirius Red and Masson’s trichrome staining.

### Danazol treatment in Mdr2^–/–^ mice.

Male and female 6-week-old Mdr2^–/–^ mice were injected i.p. with 1.6 mg/kg/d Danazol (Sigma Aldrich, catalog D8399) dissolved in 5% DMSO, 40% PEG300, 5% Tween-80, and 50% H_2_O (made fresh every day). Control mice received vehicle composed of DMSO, PEG-300, Tween-80, and H_2_O. Mice were injected 5 d/wk for 5 weeks after which the liver tissue was harvested and serum was collected for histology, mRNA, and liver biochemistry analysis with the mammalian liver profile rotor (catalog 500-7128; Abaxis) on the Vetscan VS2 (Abaxis).

### Telomere FISH.

Paraffin-embedded sections were deparaffinized in xylene (3 × 5 minutes each) followed by rehydration in ethanol (100%, 95%, 90%, 70%; 2 × 2 minutes). After PBS wash, the sections were exposed to antigen retrieval (0.1 M sodium citrate, pH 6.0) for 30 minutes. After placement for 60 minutes in 1% normal goat serum in 0.1% BSA in PBS block, the sections were incubated with γH2A.x Ab (Cell Signaling Technology, catalog 9718; 1:400 in blocking solution) overnight at 4°C. The next day, sections were incubated with biotinylated secondary Ab for 30 minutes followed by a 30-minute incubation in streptavidin Cy5 Ab (Thermo Fisher Scientific, catalog SA1011). The sections were washed 3 times in PBS, cross-linked with 4% paraformaldehyde for 20 minutes, and dehydrated in graded ethanol (90%, 95%, 100%). Sections were denatured for 10 minutes at 80°C in hybridization buffer (70% formamide [Sigma Aldrich], 25 mM MgCl_2_, 0.1 M Tris [pH 7.2], 5% blocking reagent [Roche]) containing 2.5 μg/mL Cy-3–labeled telomere-specific (CCCTAA) peptide nucleic acid probe (Panagene), followed by hybridization for 2 hours at room temperature in the dark. Slides were washed twice with 70% formamide in 2 × SSC for 15 minutes, followed by washes in 2 × SSC and PBS for 10 minutes. Sections were incubated with DAPI, mounted, and imaged. In-depth Z-stacking was used (a minimum of 40 optical slices with a ×63 objective). Relative telomere length was measured by telomere intensity per nucleus. The number of TAF per cell was assessed by quantification of partially or fully overlapping (in the same optical slice) signals from telomere probe and γH2A.X in slice-by-slice analysis.

### RNA ISH.

RNA-ISH was performed with the RNAscope protocol from Advanced Cell Diagnostics Inc. Paraffin sections were deparaffinized with xylene, rehydrated in graded ethanol (EtOH), and H_2_O_2_ was applied for 10 minutes at room temperature followed by 2 washes in H_2_O. Sections were placed in retrieval reagent and heated for 30 minutes. After washes in H_2_O and 100% EtOH, sections were air dried. Sections were treated with protease plus for 30 minutes at 40°C, washed with H_2_O, and incubated with target probe (p21 or TERT) for 2 hours at 40°C. Afterward, slides were washed with H_2_O, followed by incubation with amplifier 1 (AMP1; 30 minutes at 40°C) and next washed with wash buffer (WB) and AMP2 (15 minutes at 40°C), WB and AMP3 (30 minutes at 40°C), WB and AMP4 (15 minutes at 40°C), WB and AMP5 (30 minutes at room temperature) and WB and AMP6 (15 minutes at room temperature). Finally, the RNAscope 2.5 HD Reagent Kit-RED was used for chromogenic labeling. After counterstaining with hematoxylin, sections were mounted, a coverslip placed, and sections imaged using a Zeiss microscope. In some cases, after chromogenic labeling, sections were mounted with Prolong containing DAPI and imaged using a Zeiss fluorescence microscope. For co-immunofluorescence with RNA ISH after the chromogenic detection of the ISH signal, slides were blocked with codetection blocker (Advanced Cell Diagnostics, catalog 323170) immunostained for CK19, CK7, or 53BP1 at 1:100 using the Ab diluent (catalog 323160) and detected with Alexa fluor 488 secondary Abs.

### Western blotting.

Cells were collected and lysed in RIPA buffer with protease and phosphatase inhibitors. After centrifugation at 14,500*g* for 15 minutes, supernatants were used for protein assay using a Bio-Rad reagent. Protein (5–20 μg) was loaded onto 4% to 20% Tris-glycine gels, electrophoresed, and transferred onto nitrocellulose membranes (Scientific Laboratory Supplies) for blotting. The membrane was blocked, incubated with primary Abs (Supplemental Table 1), rinsed with TBS plus Tween, and incubated with HRP-conjugated secondary Abs. After overnight incubation at 4°C, the membranes were washed and incubated for 1 hour in appropriate secondary Ab. The blots were developed using the Kwik Quant Imager (Kindle Biosciences). Images were quantified using Fiji software (https://fiji.sc/).

### RT-PCR.

Total RNA was extracted from cells using the RNeasy Plus Mini Kit (Qiagen). Reverse transcription was performed with 500 ng of RNA using oligo (dT) primer and SuperScript III. Real-time PCR was performed in a volume of 20 μL using Sybr Green Master Mix and the 7500 Real-Time PCR System (Applied Biosystems). Primer sequences are shown in Supplemental Table 2.

### CCL2 ELISA and cytokine array.

Conditioned medium from H69 cells transfected with HA-TERT and treated with 10 ng/mL TGF-β or vehicle was tested for CCL2 levels using a CCL2 ELISA kit (R&D Systems, catalog DCP00) according to manufacturer’s instructions. The same conditioned medium was used for the XL proteome profiler (R&D Systems, catalog ARY022B) according to manufacturer’s instructions.

### ChIP-Seq.

Low-passage NHC cells, exposed to irradiation to induce senescence, or untreated control cells were fixed with 1% formaldehyde followed by glycine quench. Fixed cells were collected in PBS, washed once, and the cell pellet was sent to the Epigenomics Development Laboratory (Mayo Clinic, Rochester, Minnesota, USA) for ChIP with H3K27ac Ab. Samples were then sequenced at the Genome Analysis Core (Mayo Clinic, Rochester, Minnesota, USA). After sequencing, read quality was assessed by FastQC (51). Fastx-trimmer (52) was then used to trim reads of adapters and poor-quality bases; subsequently, these reads were mapped to the hg38 human genome using Bowtie2 (53). Samtools (54) was used to create pseudoreplicates of both control and irradiated samples. MACS2 (version 2.2.7.1) (55) was used to call narrow peaks for these data. Bedtools (56) and custom scripts were used to merge peaks and identify the highest summit within each merged peak. The intersection of coding gene promotor regions (i.e., 1,000 bp on either side of the transcriptional start site) and the merged peaks was performed to identify acetylated genes. Motif analysis via HOMER, version 4.1 (57), was performed on these peaks. ShinyGO (58), version 0.77, was run on the genes that gained acetylation, as described above, to determine KEGG (59) pathway enrichments and visualization through Pathview (60). A differential binding analysis by diffbind (61) using the union of peaks called in all pseudoreplicates was performed. Deeptools (62) was used to visualize regions across the genome.

### Patient samples.

Deidentified paraffin-embedded liver tissue sections were obtained through the Mayo Clinic Center for Cell Signaling in Gastroenterology Clinical Core. Patient demographics are included in Supplemental Table 3.

### Statistics.

Reported data represent typical experiments reproduced at least 3 times. The data were analyzed using 1-way ANOVA with Tukey’s post-test or 2-tailed *t* test, using GraphPad Prism software. The difference was considered significant when *P* < 0.05. The results are presented as mean ± SEM.

### Study approval.

All animal experiments followed protocols approved by Mayo Clinic IACUC (A5490) and are reported in accordance with ARRIVE guidelines. Patient liver explant tissues were obtained and analyzed under Mayo Clinic IRB-approved protocols (IRB 21-006846).

### Data availability.

ChIP-Seq data are deposited in the GEO database (GSE239292). All other data can be accessed in the supplemental material.

## Author contributions

NJS conceived, designed, and conducted experiments; acquired and analyzed the data; provided funding; and contributed to manuscript writing and editing the final version of the manuscript. AA conducted in vitro and in vivo experiments, acquired and analyzed data, and contributed to manuscript writing and editing the final version of the manuscript. UY and CG assisted with the in vivo experiments. AL assisted with telomere intensity and TAF analysis. AQW analyzed the ChIP-Seq data. DJ contributed to oversight of the study, data analysis, and manuscript editing. RCH supervised the study, contributed to oversight of the study, provided funding, and contributed to manuscript writing and editing. The order of shared co–first authorship was determined on the basis of contribution to the number of figures in the manuscript.

## Supplementary Material

Supplemental data

Supporting data values

## Version 1 09/14/2023

In-Press Preview

## Version 2 10/23/2023

Electronic publication

## References

[1] Lazaridis KN, LaRusso NF (2016). Primary sclerosing cholangitis. N Engl J Med.

[2] Chung BK (2018). Cholangiocytes in the pathogenesis of primary sclerosing cholangitis and development of cholangiocarcinoma. Biochim Biophys Acta Mol Basis Dis.

[3] Tabibian JH (2014). Cholangiocyte senescence by way of N-ras activation is a characteristic of primary sclerosing cholangitis. Hepatology.

[4] Meadows V (2021). Biliary epithelial senescence in liver disease: there will be SASP. Front Mol Biosci.

[5] Cazzagon N (2021). Cholangiocyte senescence in primary sclerosing cholangitis is associated with disease severity and prognosis. JHEP Rep.

[6] Karlsen TH (2017). Primary sclerosing cholangitis — a comprehensive review. J Hepatol.

[7] Aseem SO (2021). Epigenomic evaluation of cholangiocyte transforming growth factor-β signaling identifies a selective role for histone 3 lysine 9 acetylation in biliary fibrosis. Gastroenterology.

[8] Jalan-Sakrikar N (2019). Proteasomal degradation of enhancer of zeste homologue 2 in cholangiocytes promotes biliary fibrosis. Hepatology.

[9] Kumari R, Jat P (2021). Mechanisms of cellular senescence: cell cycle arrest and senescence associated secretory phenotype. Front Cell Dev Biol.

[10] Gorgoulis V (2019). Cellular senescence: defining a path forward. Cell.

[11] Tchkonia T (2013). Cellular senescence and the senescent secretory phenotype: therapeutic opportunities. J Clin Invest.

[12] Kirkland JL, Tchkonia T (2017). Cellular senescence: a translational perspective. EBioMedicine.

[13] Jalan-Sakrikar N (2022). Induced pluripotent stem cells from subjects with primary sclerosing cholangitis develop a senescence phenotype following biliary differentiation. Hepatol Commun.

[14] Bogert PS (2022). Cellular senescence in the cholangiopathies. Curr Opin Gastroenterol.

[15] Greider CW (1998). Telomeres and senescence: the history, the experiment, the future. Curr Biol.

[16] Aini W (2014). Accelerated telomere reduction and hepatocyte senescence in tolerated human liver allografts. Transpl Immunol.

[17] d’Adda di Fagagna F (2003). A DNA damage checkpoint response in telomere-initiated senescence. Nature.

[18] Sasaki M (2008). Telomere shortening in the damaged small bile ducts in primary biliary cirrhosis reflects ongoing cellular senescence. Hepatology.

[19] Penrice DD (2023). Telomere dysfunction in chronic liver disease: The link from aging. Hepatology.

[20] Rudolph KL (2000). Inhibition of experimental liver cirrhosis in mice by telomerase gene delivery. Science.

[21] Lin S (2018). Distributed hepatocytes expressing telomerase repopulate the liver in homeostasis and injury. Nature.

[22] Ogrodnik M (2017). Cellular senescence drives age-dependent hepatic steatosis. Nat Commun.

[23] Liu T (2019). Telomerase reverse transcriptase ameliorates lung fibrosis by protecting alveolar epithelial cells against senescence. J Biol Chem.

[24] Huda N (2022). Telomere length in patients with alcohol-associated liver disease: a brief report. J Investig Med.

[25] Carulli L (2015). Telomere shortening as genetic risk factor of liver cirrhosis. World J Gastroenterol.

[26] Laish I (2016). Telomere dysfunction in nonalcoholic fatty liver disease and cryptogenic cirrhosis. Cytogenet Genome Res.

[27] Shin HK (2021). Association between telomere length and hepatic fibrosis in non-alcoholic fatty liver disease. Sci Rep.

[28] Laish I (2015). Telomere dysfunction in peripheral blood lymphocytes from patients with primary sclerosing cholangitis and inflammatory bowel disease. Dig Liver Dis.

[29] Tabibian JH (2014). Characterization of cultured cholangiocytes isolated from livers of patients with primary sclerosing cholangitis. Lab Invest.

[30] Hewitt G (2012). Telomeres are favoured targets of a persistent DNA damage response in ageing and stress-induced senescence. Nat Commun.

[31] Bernadotte A (2016). Markers of cellular senescence. Telomere shortening as a marker of cellular senescence. Aging (Albany NY).

[32] Fickert P (2007). A new xenobiotic-induced mouse model of sclerosing cholangitis and biliary fibrosis. Am J Pathol.

[33] Townsley DM (2016). Danazol treatment for telomere diseases. N Engl J Med.

[34] Bryan C (2015). Structural basis of telomerase inhibition by the highly specific BIBR1532. Structure.

[35] Shim HS (2021). Telomerase reverse transcriptase preserves neuron survival and cognition in alzheimer’s disease models. Nat Aging.

[36] Wu CH (2007). Cellular senescence is an important mechanism of tumor regression upon c-Myc inactivation. Proc Natl Acad Sci U S A.

[37] Vega-Benedetti AF (2018). *PLAGL1* gene function during hepatoma cells proliferation. Oncotarget.

[38] Gal C (2021). DREAM represses distinct targets by cooperating with different THAP domain proteins. Cell Rep.

[39] Hartmann D (2011). Telomerase gene mutations are associated with cirrhosis formation. Hepatology.

[40] Calado RT (2011). Constitutional telomerase mutations are genetic risk factors for cirrhosis. Hepatology.

[41] Jaskelioff M (2011). Telomerase reactivation reverses tissue degeneration in aged telomerase-deficient mice. Nature.

[42] Chakravarti D (2020). Telomere dysfunction activates YAP1 to drive tissue inflammation. Nat Commun.

[43] Bar C, Blasco MA (2016). Telomeres and telomerase as therapeutic targets to prevent and treat age-related diseases. F1000Res.

[44] Bernardes de Jesus B (2012). Telomerase gene therapy in adult and old mice delays aging and increases longevity without increasing cancer. EMBO Mol Med.

[45] Bernardes de Jesus B, Blasco MA (2013). Telomerase at the intersection of cancer and aging. Trends Genet.

[46] Ding D (2013). Human telomerase reverse transcriptase regulates MMP expression independently of telomerase activity via NF-κB-dependent transcription. FASEB J.

[47] Ponnala S (2011). MMP-9 silencing regulates hTERT expression via β1 integrin-mediated FAK signaling and induces senescence in glioma xenograft cells. Cell Signal.

[48] Haendeler J (2009). Mitochondrial telomerase reverse transcriptase binds to and protects mitochondrial DNA and function from damage. Arterioscler Thromb Vasc Biol.

[49] Gild P (2018). Liver disease in men undergoing androgen deprivation therapy for prostate cancer. J Urol.

[50] Donati B, Valenti L (2016). Telomeres, NAFLD and chronic liver disease. Int J Mol Sci.

[51] Kocarnik JM (2010). Cancer incidence, mortality, years of life lost, years lived with disability, and disability-adjusted life years for 29 cancer groups from 2010 to 2019: a systematic analysis for the Global Burden of Disease Study 2019. JAMA Oncol.

[52] http://hannonlab.cshl.edu/fastx_toolkit/.

[53] Langmead B, Salzberg SL (2012). Fast gapped-read alignment with Bowtie 2. Nat Methods.

[54] Danecek P (2021). Twelve years of SAMtools and BCFtools. Gigascience.

[55] Zhang Y (2008). Model-based analysis of ChIP-Seq (MACS). Genome Biol.

[56] Quinlan AR, Hall IM (2010). BEDTools: a flexible suite of utilities for comparing genomic features. Bioinformatics.

[57] Heinz S (2010). Simple combinations of lineage-determining transcription factors prime cis-regulatory elements required for macrophage and B cell identities. Mol Cell.

[58] Ge SX (2020). ShinyGO: a graphical gene-set enrichment tool for animals and plants. Bioinformatics.

[59] Kanehisa M, Sato Y (2020). KEGG Mapper for inferring cellular functions from protein sequences. Protein Sci.

[60] Luo W, Brouwer C (2013). Pathview: an R/Bioconductor package for pathway-based data integration and visualization. Bioinformatics.

[61] Ross-Innes CS (2012). Differential oestrogen receptor binding is associated with clinical outcome in breast cancer. Nature.

[62] Ramirez F (2016). deepTools2: a next generation web server for deep-sequencing data analysis. Nucleic Acids Res.

