# Supplementary material for: Telomere dysfunction promotes cholangiocyte senescence and biliary fibrosis in primary sclerosing cholangitis
Source: JCI Insight. 2023 Oct 23;8(20):e170320. doi: 10.1172/jci.insight.170320 (PMC10619490; doi:10.1172/jci.insight.170320)
Supplement: Supplemental data [file jciinsight-8-170320-s184.pdf]

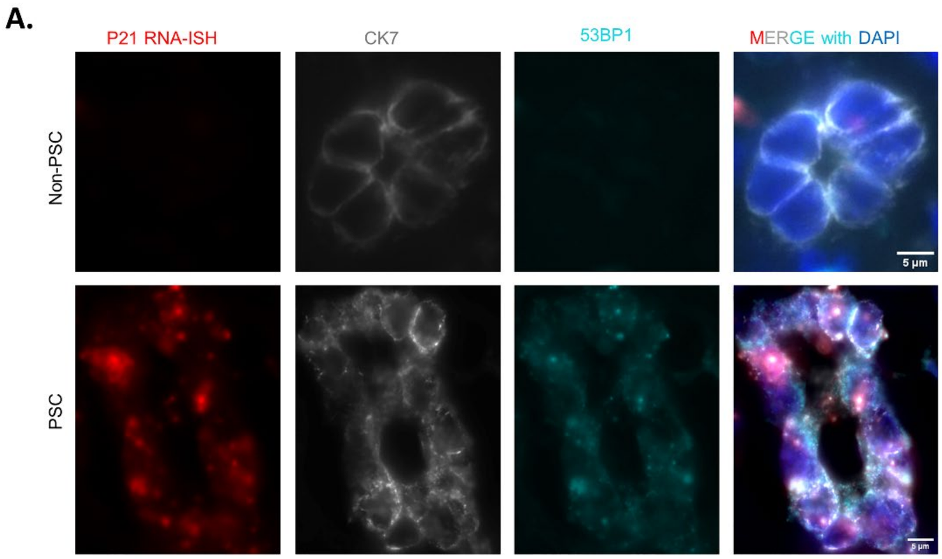


**Supplemental Figure 1**

**Supplementary FIG. 1.** (A) p21 RNA-ISH (red) with co-immunofluorescence for bile duct marker, CK7 (gray) and DNA damage marker (53BP1) on non-PSC and PSC liver tissues showing co-localization of p21 and 53BP1 in cholangiocytes.

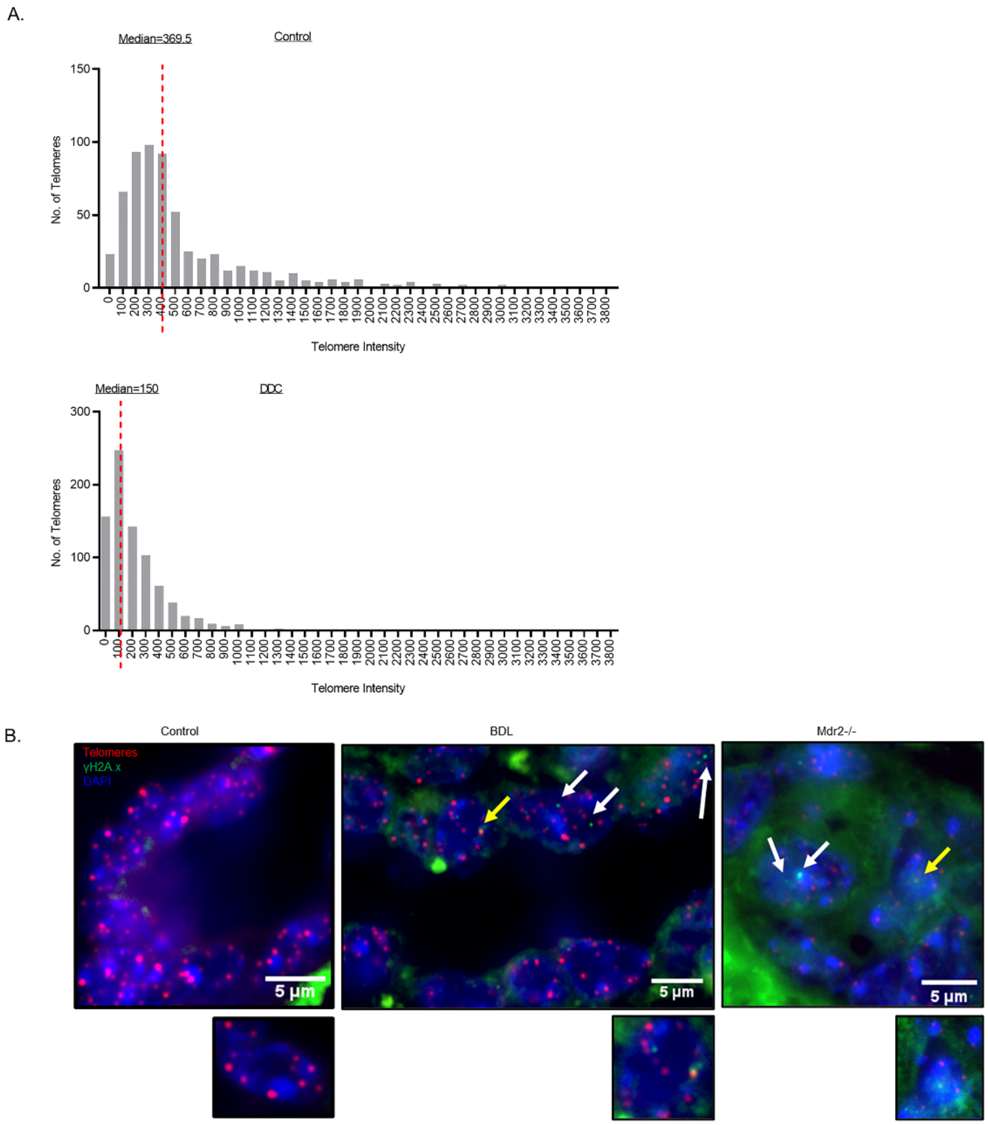


**Supplemental Figure 2**

**Supplementary FIG. 2.** (A) Distribution of telomere intensity in chow-fed mice (top) vs. DDC-fed mice (bottom) indicates increased prevalence of short telomeres in DDC liver tissue. (B) Immuno-FISH for telomeres and ɣH2A.x on liver tissue from bile-duct ligated (BDL) mice and MDR2-/- mice reveals increased telomere-associated DNA damage foci and reduced telomere length (intensity) compared to control mice.

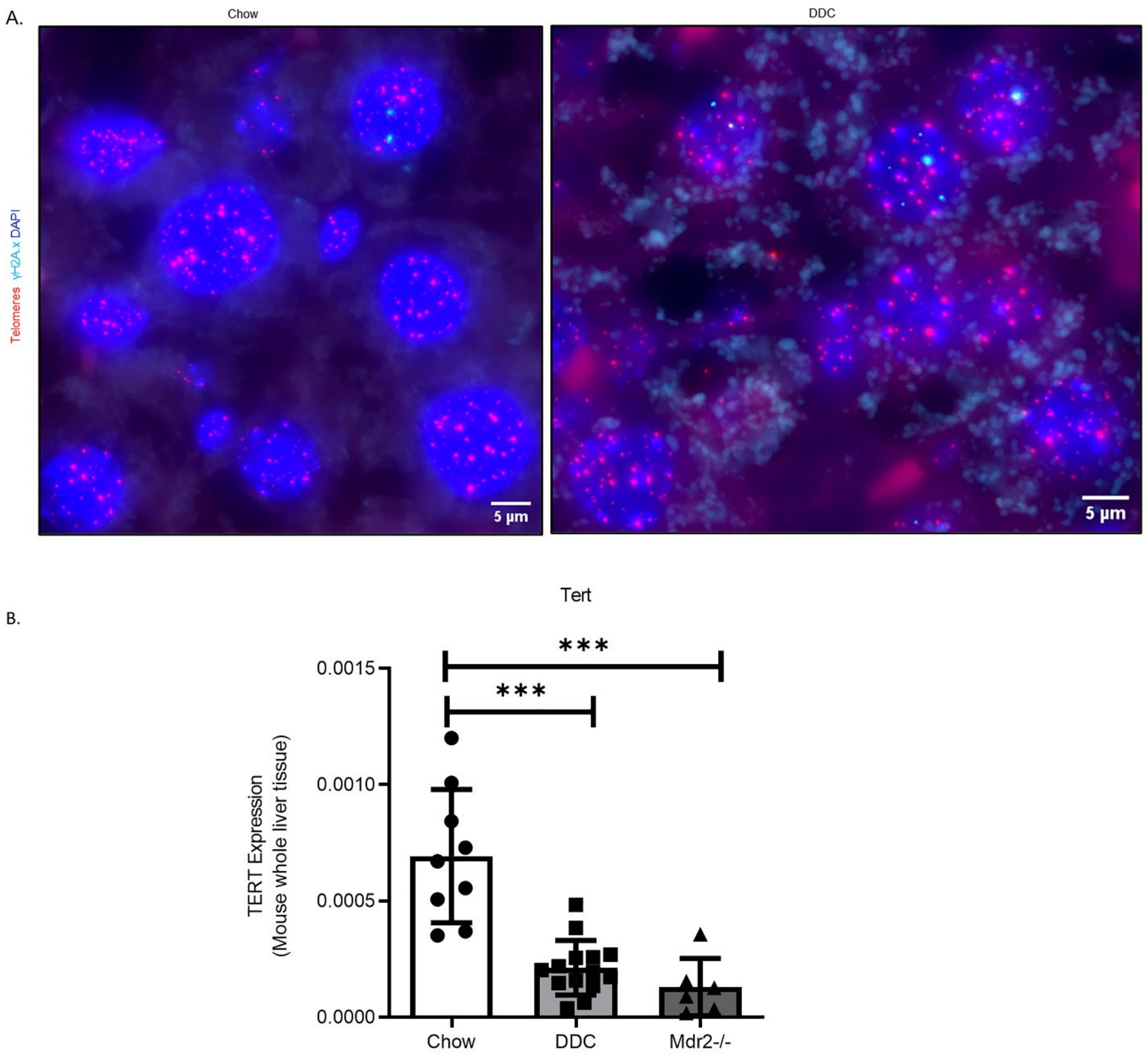


**Supplemental Figure 3**

**Supplementary FIG. 3.** (A) Telomere FISH (red) with DNA damage marker, γH2A.x on chow and DDC-fed mice liver tissues demonstrating some increase in DNA damage in hepatocytes with DDC-mediated injury but not significant change in telomere intensity. (B) mRNA analysis on whole liver tissue displays significant reduction in TERT mRNA in DDC-fed and MDR2-/- mice compared to control mice. ***P < 0.0001, ****P < 0.00001. All error bars are SEM, n = 6-14 animals per group.

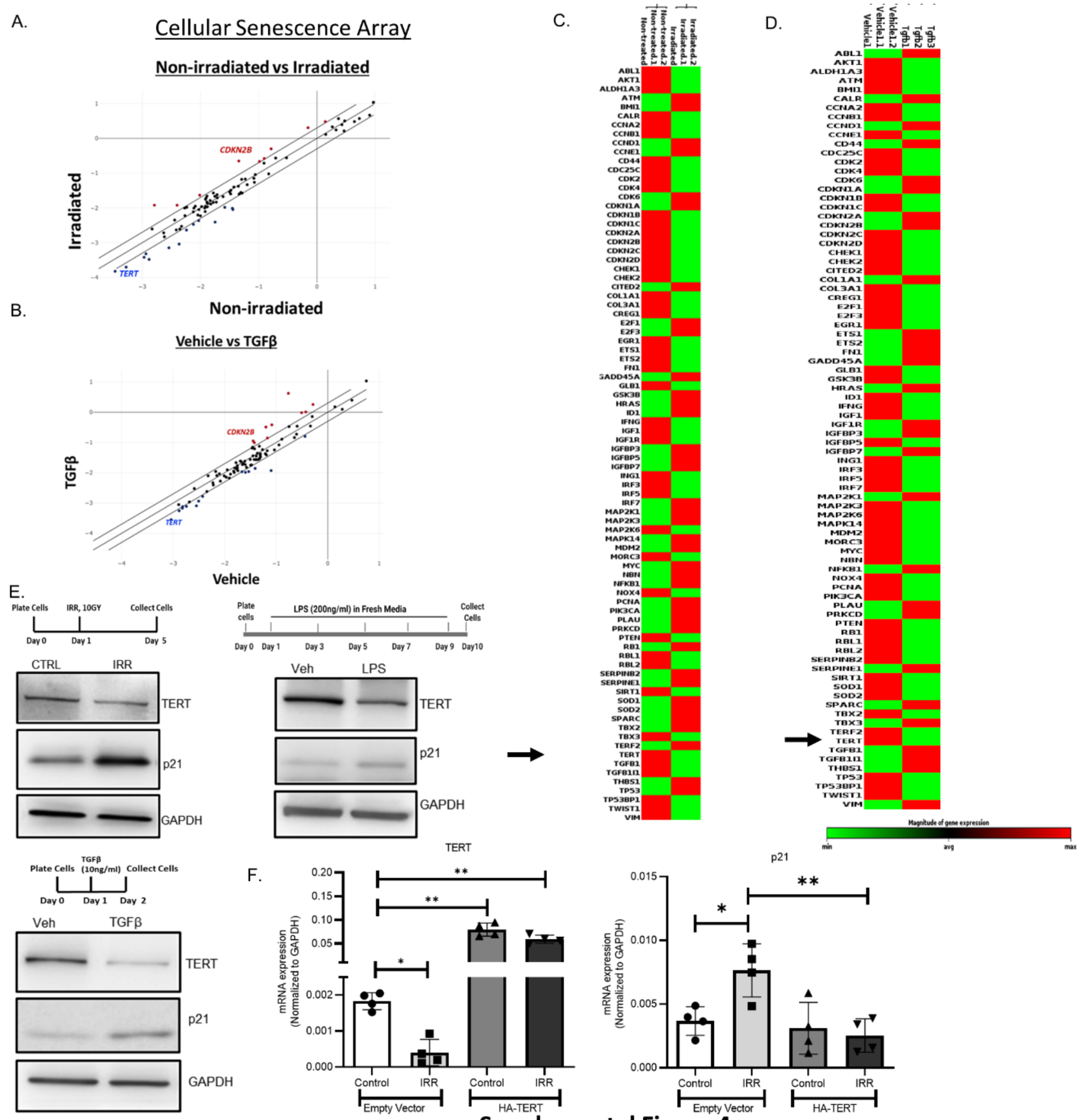


**Supplemental Figure 4**

**Supplementary FIG. 4.** (A) Cellular senescence array on HiBEC revealing reduction in TERT levels in cells exposed to irradiation compared to non-irradiated cells. (B) Cellular senescence array on H69 cholangiocytes treated with 10ng/mL TGFβ (C) Map representing gene changes in HiBEC cell line exposed to irradiation (green: up-regulated gene set; red: down-regulated gene set). n = 3. (D) Map representing gene changes in H69 cells treated with 10 ng/mL TGF-β for 24 hours confirming downregulation in TERT and upregulation of p21 (green: up-regulated gene expression; red: down-regulated gene expression). n = 3. (E) Western blots of HiBEC cholangiocytes exposed to irradiation (left), 200ng/mL LPS (right), or 10ng/mL TGF-β (bottom) confirms reduction in TERT and increase in p21 compared to control cells. (F) RT-PCR analysis for TERT and p21 in HiBEC cells transfected with HA-TERT followed by exposure to irradiation. One-Way ANOVA followed by Tukey’s post test *P < 0.01, **P<0.001. All error bars are SEM, n = 4.

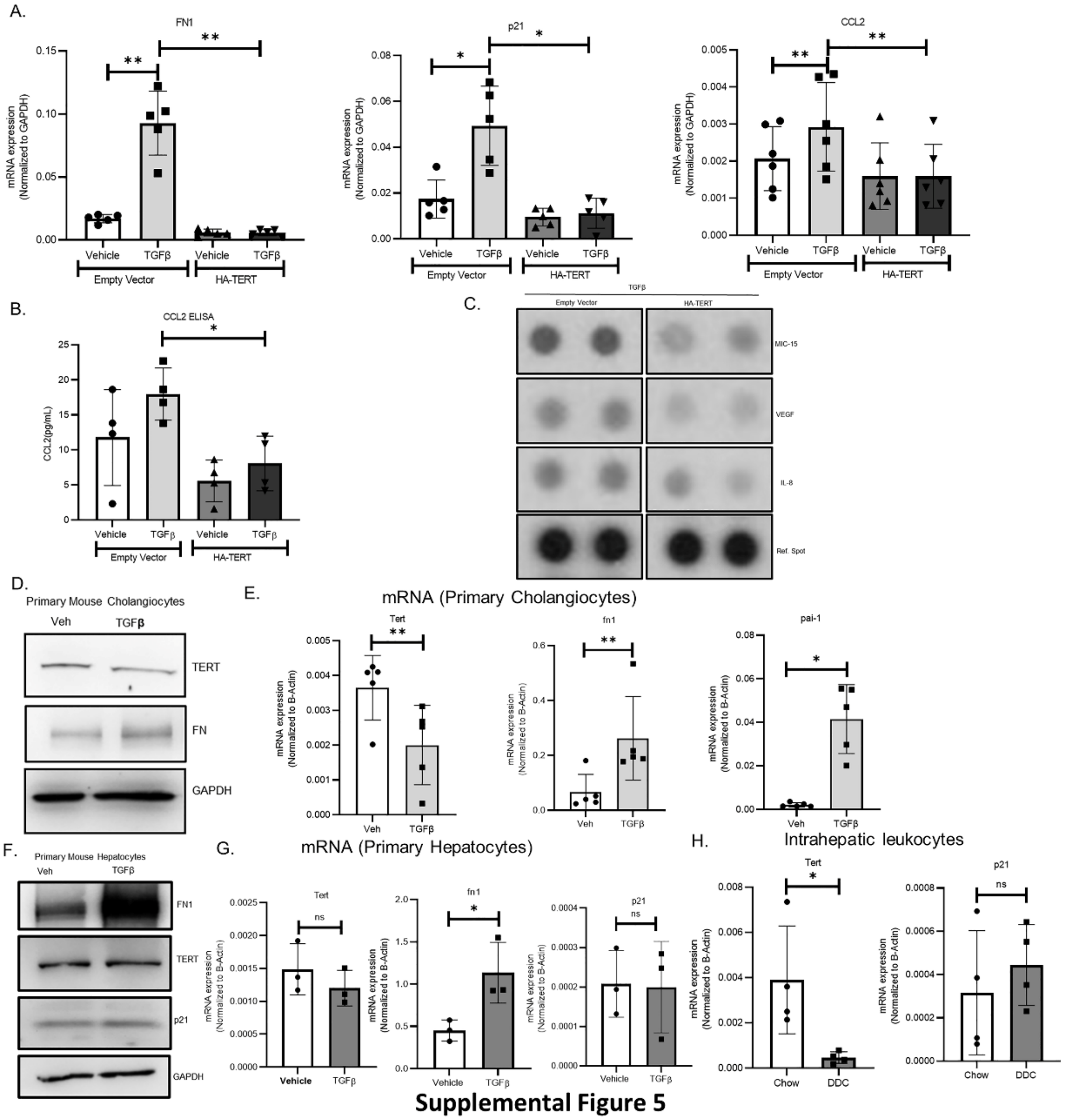


**Supplemental Figure 5**

**Supplementary FIG. 5.** (A) mRNA analysis in H69 cells treated with 10 ng/mL TGF-β for 24 hours demonstrating attenuation of HSC activator, FN1; senescent marker, p21, and cytokine, CCL2 in cholangiocytes transfected with HA-TERT. $*P < 0.01$. All error bars are SEM, n = 5. (B) Enzyme-Linked Immunosorbent Assay (ELISA) for CCL2 on conditioned media of TGFβ-treated cholangiocytes reveals attenuated secretion upon TERT overexpression. $*P < 0.01$. All error bars are SEM, n = 4. (C) XL Cytokine Array (ARY022B) performed on the secretome of H69 cells treated with 10 ng/mL TGFβ displays reduced production of proinflammatory cytokines MIC-15, VEGF, and IL-8, from cholangiocytes overexpressing TERT. (D) Immunoblot analysis on primary mouse cholangiocytes treated with 10 ng/mL TGF-β for TERT and FN1 with GAPDH as loading control. Cells treated with TGF-β show reduced TERT and concurrent increase in FN compared to vehicle. (E) RT-PCR analysis of ki-67, tert, pai-1, and fn1 from primary mouse cholangiocytes treated with 10ng/mL TGF-β. $*P < 0.01$, $***P < 0.0001$. All error bars are SEM, n = 4-6 animals per group. (F) Immunoblotting of protein lysates from primary mouse hepatocytes treated with vehicle or 10ng/mL TGFβ shows augmented FN1, but no alterations in TERT or p21 protein levels with TGFβ stimulation. (G) RT-PCR on primary hepatocytes demonstrates amplified expression of fn1 and no significant change in tert and p21 gene expression. $*P < 0.01$. All error bars are SEM, n = 3. (H) mRNA analysis on intrahepatic leukocytes shows decreased TERT expression, but no significant difference in p21 mRNA levels in DDC-fed mice compared to chow-fed mice $*P < 0.01$. All error bars are SEM, n =4.

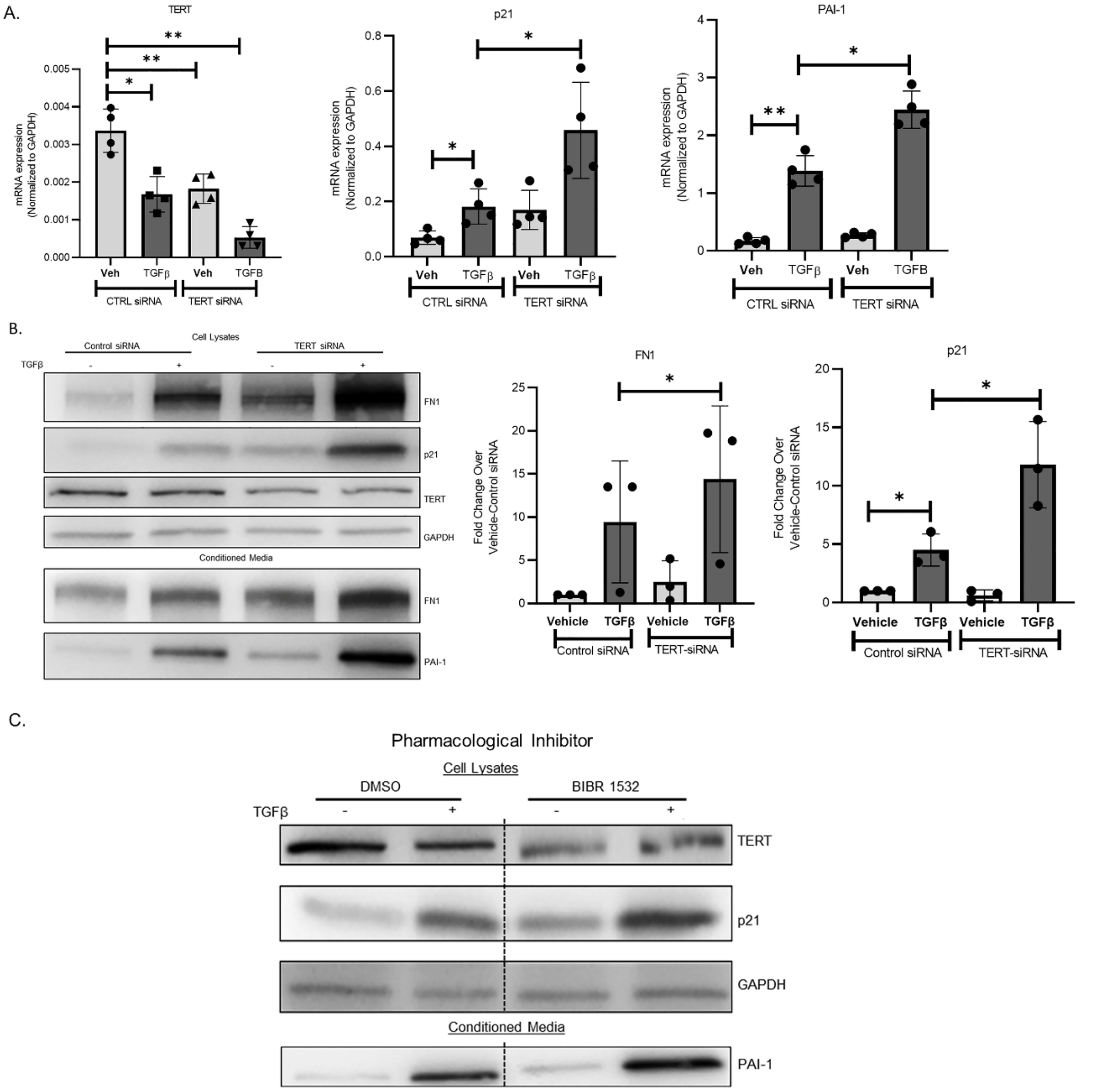


**Supplementary Figure 6**

**Supplementary FIG. 6.** (A) RT-PCR analysis on H69 cells incubated with control or TERT siRNA demonstrates reduction of TERT transcript and amplification of TGFβ-induced p21 and PAI-1 gene expression upon TERT knockdown. *P < 0.01, *P < 0.001. All error bars are SEM, n = 3. (B) Immunoblot analysis reveals TERT knockdown with siRNA in H69 cholangiocytes exacerbates TGFβ-mediated p21 and FN1 (top, quantification on right) as well as release of FN1 and PAI-1 in the media (bottom). P < 0.01. All error bars are SEM, n = 3. (C) H69 cells were exposed to DMSO or TERT inhibitor, BIBR 1532, followed by 24-hour treatment with 10 ng/mL TGFβ. Cell lysates and conditioned media were immunoblotted revealing exacerbated TGFβ-mediated p21 and PAI-1 levels, respectively, compared to DMSO-treated cholangiocytes, the lanes were run on the same gel but were noncontiguous.

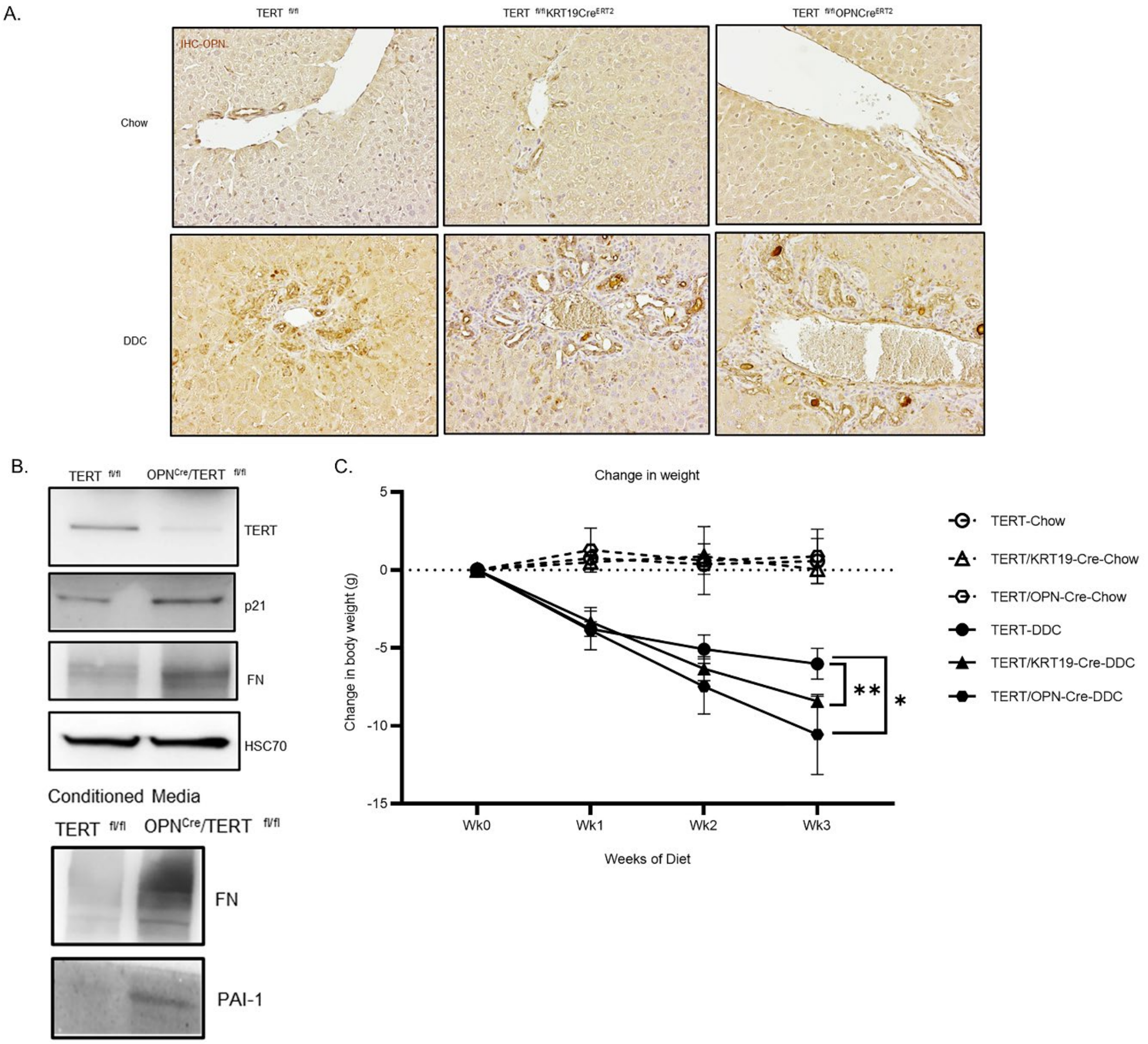


**Supplemental Figure 7**

**Supplementary FIG. 7.** (A) Immunohistochemistry for osteopontin (Opn) in bile ducts from TERT$^{fl/fl}$, Opn Cre$^{ERT2}$/ Tert$^{fl/fl}$, and Krt9 Cre$^{ERT2}$/ Tert$^{fl/fl}$ liver tissue. (B) Western blotting on lysates and conditioned media (bottom) of primary cholangiocytes from Tert$^{fl/fl}$ or Opn Cre$^{ERT2}$/ Tert$^{fl/fl}$ showing decreased TERT with concurrent increase in p21, FN, and PAI-1. (C) Change in body weight of mice on chow or DDC diet revealing enhanced weight loss upon TERT deletion in DDC-fed mice. *$P < 0.01$, **$P < 0.001$. All error bars are SEM, n=4-5 animals per group.

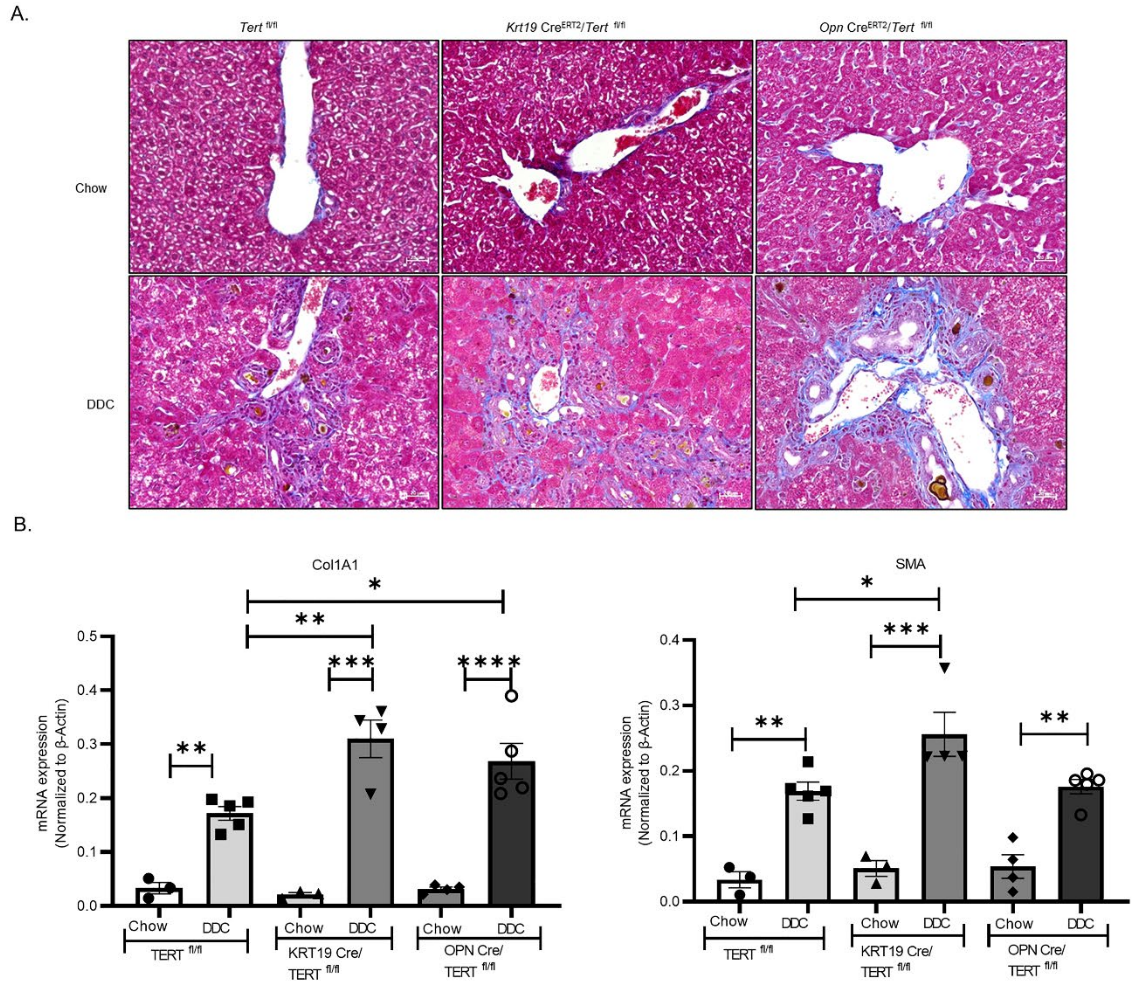


**Supplemental Figure 8**

**Supplementary FIG. 8.** (A) Masson's Trichrome staining of chow or DDC-fed mice shows intensified fibrosis in mice lacking TERT compared to control mice on DDC diet. (B) mRNA expression of fibrogenic markers, Col1A1 (left) and SMA (right), in chow and DDC-fed mice displaying increased fibrosis in mice lacking TERT. *P < 0.01. **P < 0.001, ***P < 0.0001,****P < 0.00001.All error bars are SEM, n = 3-5 animals per group.

**Supplementary Tables**

*Supplementary Table 1: Primary Antibodies*

| Antibody | Supplier/Catalog No. |
|---|---|
| FN1 | BD Biosciences #610078 |
| TERT | Novus Biologicals #NB100-297<br>Santa Cruz #377511 |
| PAI-1 | Santa Cruz #8979 |
| HSC70 | Santa Cruz #sc-7298 |
| α-SMA | Abcam #ab5694 |
| P21 | Abcam #ab188224 |
| γH2A.X | Cell Signaling #9718 |
| GAPDH | Invitrogen #AM4300 |
| 53BP1 | Cell Signaling #4937 |
| OPN | R&D Systems #AF808 |
| HA | Millipore Sigma #12CA5 |

*Supplementary Table 2: RT-PCR primers sequences*

| Gene | Forward | Reverse |
|---|---|---|
| hP21 | TGTCACTGTCTTGTACCCTTG | GGCGTTTGGAGTGGTAGAA |
| hTERT | GCACGGCTTTTGTTCAGATG | CGGTTGAAGGTGAGACTGG |
| hFN1 | GATAAATCAACAGTGGGAGC | CCCAGATCATGGAGTCTTTA |
| hGAPDH | | |
| mTERT | TCTTGCGGTTGAAGTGTCAC | TCCTAACACGCTGGTCAAAG |
| mFN1 | CTTTGGCAGTGGTCATTTCAG | ATTCTCCCTTTCCATTCCCG |
| mKi-67 | TGCCCGACCCTACAAAATG | GAGCCTGTATCACTCATCTGC |
| mPAI-1 | TGCAAAAGGTCAGGATCGAG | ATTGTCTCTGTCGGGTTGTG |
| mCol1A1 | GAGCGGAGAGTACTGGATCG | GCTTCTTTTCCTTGGGGTTC |
| mSMA | AAACAGGAATACGACGAAG | CAGGAATGATTTGGAAAGGA |
| mβ-Actin | AGAGGGAAATCGTGCGTGAC | CAATAGTGATGACCTGGCCGT |

*Supplementary Table 3: Patient Demographics used in the study.*

| De-Identified Number | Age/Sex | PSC Stage | Sample | Histology Notes |
|---|---|---|---|---|
| 10008542 | 44/M | 1 | Needle Biopsy | Underlying Ulcerative Colitis (UC), ceroid-laden macrophages |
| 10008543 | 53/M | 1 | Needle Biopsy | No UC |
| 10008545 | 64/F | 2 | Needle Biopsy | No UC |
| 10008548 | 38/M | 3 | Explant | UC |
| 10008565 | 39/M | 2 | Wedge Biopsy | UC |
| 10008564 | 69/F | 2 | Wedge Biopsy | UC |
| 10008532 | 46/M | 4 | Explant | |
| 11013784 | 46/M | Unknown | Explant | UC |
| 11013779 | 35/M | Unknown | Explant | UC |
| 11013782 | 67/M | Unknown | Explant | UC |
| Deceased Donor tissue | Unknown/M | Non-PSC | Donor Tissue | |
| Deceased Donor tissue | Unknown/M | Non-PSC | Donor Tissue | |
| Deceased Donor tissue | Unknown/M | Non-PSC | Donor Tissue | |
| Deceased Donor tissue | Unknown/F | Non-PSC | Donor Tissue | |
| Deceased Donor tissue | Unknown/F | Non-PSC | Donor Tissue | |
